# Genetics, Not Weather, Informs Migration Behaviors in a Long‐Distance Migrant Songbird

**DOI:** 10.1002/ece3.74245

**Published:** 2026-09-26

**Authors:** Kathryn E. McGee, Steven E. Travis, Allan M. Strong, Noah G. Perlut

**Affiliations:** ^1^ School of Biological Sciences University of New England Biddeford Maine USA; ^2^ Rubenstein School of Environment and Natural Resources University of Vermont Burlington Vermont USA; ^3^ School of Marine and Environmental Programs University of New England Biddeford Maine USA

**Keywords:** Bobolink, carry‐over effects, *Dolichonyx oryzivorus*, full life‐cycle, geolocator, grassland songbird, microsatellites

## Abstract

Migration is the riskiest portion of the annual breeding cycle, with long‐distance migratory birds enduring long periods of varied landscapes, dangerous weather, and geographical barriers such as long stretches of open water. Therefore, birds utilize a combination of both innate (i.e., genetic) and environmental cues to maximize their chances of survival during migration. Studies have suggested that short‐distance migrants are more likely to adjust migration timing to match non‐endogenous cues like local weather conditions, while long‐distance migrants rely heavily on genetically‐ingrained behaviors to determine the start of spring migration. While many studies have examined the effects of environmental variables and genetics individually, few have examined the combined effects of these influences throughout the annual cycle of a migratory songbird species. We examined the correlation of both genetics and the environmental conditions experienced by a long‐distance migrant, the Bobolink ( *Dolichonyx oryzivorus* ), with the timing and latitude of seven migratory events throughout their annual cycle. To our knowledge, this is the first study to document the possible effects of genetic relatedness throughout the annual cycle of a long‐distance migratory songbird using neutral genetic markers. Genetic relatedness was highly correlated with the timing (4 of 7) rather than latitude (1 of 7) of migration events. Departure from pre‐water crossing stages, arrival to wintering grounds and departure from South America were the only migration events in the annual cycle where neither the timing nor location of the event were correlated with genetic relatedness. While weather had significant effects at several points in the annual cycle, these trends were often correlated with differences in latitude or timing between males and females. This study provides direct support for the long‐held theory that long‐distance migrants largely rely on innate behavior instead of environmental conditions to inform migration decisions.

## Graphical abstract

We examined the influence of both genetics and the environmental conditions experienced by a long‐distance migrant, the Bobolink ( *Dolichonyx oryzivorus* ), on the timing and latitude of seven migratory events throughout their annual cycle. Genetic relatedness largely determined the timing (4 of 7) rather than latitude (1 of 7) of migration events. This study provides direct support for the long‐held theory that long‐distance migrants largely rely on innate behavior instead of environmental conditions to inform migration decisions.

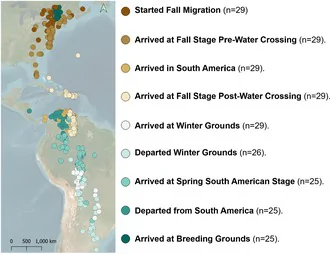

## 1 Introduction

Historically, bird migration studies have investigated a species' migratory behavior through indirect means such as arrival and departure from breeding grounds, capture along migration route at established banding stations, or stable isotope analysis (Webster et al. 2002; Helm and Liedvogel 2024). However, since Stutchbury et al. (2009), the miniaturization of tracking technologies has allowed us to unravel the intricacies of behavior throughout the entire annual cycle. Directly tracking both migration route and timing can illuminate variation that may be influenced by selection pressures to avoid risk (Fraser et al. 2013). Migration has the highest mortality rates of any period in the annual cycle (Sillett and Holmes 2002; Klaassen et al. 2014), introducing challenges such as exhaustion, the need to refuel at stopovers, predation, and changing weather conditions (Newton 2007; Goymann et al. 2010). Furthermore, many long‐distance flights cross barriers such as deserts or oceans, where landing is impossible (Fraser et al. 2013; Klaassen et al. 2014).

To prepare for migration, birds undergo a number of physiological and behavioral changes that help maximize their ability to survive periods of sustained flight. Pre‐migration, birds often gorge themselves to increase their fat stores (Goymann et al. 2010) and increase their nocturnal activity to take advantage of the safer and cooler night flying conditions (Gwinner 1996). Endogenous clocks, such as circadian rhythms, are suspected to play a large role in controlling the timing of these behavioral changes, particularly in long‐distance migrants (Gwinner 1996; Butler 2003; Marra et al. 2005). However, studies that have attempted to identify individual circadian clock genes that explain variation in migration behavior have had mixed results. In a review of circadian clock genes and migratory timing, Le Clercq et al. (2023) found an association between *Clock* gene variation and autumn migration, as well as between *Adcyap1* gene variation and spring migration. However, neither of these candidate genes was distinguishable between migratory and sedentary populations (Le Clercq et al. 2023).

Comparatively, control of migratory direction as a heritable and genetically controlled phenotype has long been established in classic cross‐breeding experiments of Zugenruhe, or migrational restlessness (Helbig 1991; reviewed by Merlin and Liedvogel 2019). European Blackcaps ( *Sylvia atricapilla* ) displayed codominant inheritance of migration direction within crossed generations, displaying a migratory phenotype intermediate to two parental populations. Blackcap populations failed to show genetic differentiation between populations using neutral markers; however, there was evidence that directional selection of males may have driven asymmetrical gene flow between the two populations (Wesołowski 2011). Similarly, Sokolovskis et al. (2023) identified two coding loci that were dominantly inherited and correlated with migratory path direction in two subspecies of a long‐distance migrant. The variation between the dominant and co‐dominant inheritance patterns observed in Sokolovskis et al. (2023) and Helbig (1991) suggests that the role of genetics in migration path is species‐specific and may be influenced by external factors.

Understanding the extent to which genetics influences migration both spatially and temporally, and how those behaviors may be inherited, is vital to understanding conservation challenges. Birds with genetically‐coded cues for initiating migration may face problems in the context of climate change, where environmental changes may happen faster than microevolution can keep up (Møller et al. 2008; Charmantier and Gienapp 2014), resulting in a phenological mismatch where birds fail to optimally utilize local food or habitat resources (Saino et al. 2010). These consequences of endogenous migratory behaviors may be present in both the spatial and temporal aspects of migration. If genetics controls the spatial aspects of migration events, changes to habitat availability along the migration route may disrupt staging activities that are vital to migratory success (Wang et al. 2022).

Bobolinks ( *Dolichonyx oryzivorus* ) are long‐distance migratory songbirds that travel ~20,000 km round trip between their breeding grounds in North America and wintering grounds in South America (Renfrew et al. 2015). Bobolinks currently have an estimated global population of 10 million individuals, with an anticipated population decrease of 50% by 2069 (Partners in Flight 2021). As a grassland obligate, habitat loss is one of the largest threats to this species (Renfrew et al. 2015). Bobolinks commonly inhabit large agricultural fields while both wintering and breeding, leaving them susceptible to changes in agricultural practices (Perlut et al. 2006; Renfrew et al. 2015). In Vermont, farmers adjusted to climate change by advancing their haying dates by 10 days, but Bobolinks did not change their nest initiation dates (McGowan et al. 2021). This misalignment has caused management conflicts where new haying schedules increasingly overlap with nesting periods (Perlut et al. 2006), further threatening the declining populations.

Numerous studies, particularly of short‐distance migrants, have shown a trend of advanced spring migration phenology as a response to climate change (Butler 2003; Usui et al. 2016; Horton et al. 2019). Plant phenology, weather, and food availability on wintering grounds may act as more reliable cues for short distance migrants than long‐distance migrants, whose wintering grounds have greater phenological separation (Butler 2003; Marra et al. 2005; Youngflesh et al. 2021). While long‐distance migrants' endogenous rhythms determine the beginning of their migration, variation in the choice of wintering grounds, speed, route, and stopover locations commonly introduce uncertainty into migration success (Marra et al. 2005; Stanley et al. 2012; Fraser et al. 2013; Helm and Liedvogel 2024). One Bobolink male tracked for two full breeding cycles showed low route repeatability, but consistency in timing at the start of both migrations (Renfrew et al. 2019). Plasticity within route choice could therefore indicate behavioral responses to environmental factors (Renfrew et al. 2019); however, long‐distance migrants are less likely to alter migration in response to vegetation phenology (Youngflesh et al. 2021).

To create effective management plans for long‐distance migrants, it is vital to understand where in the annual cycle a species is experiencing endogenous controls and where they can exhibit plasticity. There are carryover effects within the annual cycle, such as the conditions on the wintering grounds affecting breeding success later in the year (Webster et al. 2002; Saino et al. 2007). We examined the annual cycle of Bobolinks to begin to shed light on whether the extent to which the timing and location of major migratory events may be controlled by genetically‐influenced endogenous rhythms, and to what extent behavioral plasticity allows them to be influenced by carryover effects from previous stages and weather conditions. We predicted that both the start and end migration dates and locations would be significantly correlated with genetics, while stopover timing and location during migration would be largely correlated with weather conditions.

## 2 Methods

### 2.1 Study Site and Field Methods

We studied Bobolinks breeding at Shelburne Farms, Chittenden County, Vermont (44.395168, −73.247001), located in the Champlain Valley, which has 146,000 ha of managed grasslands (NASS 2012), 70% of which are actively managed during the Bobolink breeding season. The capture sites included hayfields managed by a variety of mowing schemes (Perlut et al. 2006). Adult birds were captured in 12‐m mist nets using song playback or by placing nets around active nests. Each adult bird was given a metal US Geological Survey band and three color bands. Blood samples (~50 μL) were collected from the brachial vein and stored on filter paper discs. Light‐level geolocators (0.65 g, Intigeo‐P65C2‐7‐COOL, Migrate Technology) were deployed on adult birds with a leg loop harness (Renfrew et al. 2013) crafted from 0.8 mm elastic cord. Between 2008 and 2022, a total of 306 adult birds were deployed with geolocators and in 2009–2023, 43 geolocators were successfully retrieved. For this study, we only included birds with blood samples (*n* = 29) and excluded second migration tracks from birds that successfully carried geolocators for multiple years (Table 1). Appropriate animal care was approved under the University of New England protocol IACUC‐UNE010‐2009, and banding under U.S. federal permit #23540.

**TABLE 1:** Geolocator track sample sizes per year by sex.

Year of track	Number of males	Number of females	Total
2009–2010	1	0	1
2010–2011	2	0	2
2013–2014	1	1	2
2014–2015	1	0	1
2015–2016	3	3	6
2016–2017	1	1	2
2017–2018	0	0	0
2018–2019	5	4	9
2019–2020	2	0	2
2020–2021	0	0	0
2021–2022	2	0	2
2022–2023	2	0	2
Total	20	9	29

### 2.2 Geolocator Analysis

Our geolocator analysis followed the methods described in Perlut (2018), using the BAStag package (Wotherspoon et al. 2016) in R (version 4.2.3) to calculate all twilights by date recorded by the geolocators using a light threshold of 1.5. Twilight data were converted to location using median location estimates from the package FLightR (Rakhimberdiev and Saveliev 2016; Rakhimberdiev et al. 2017). Our geolocator latitudinal error averaged 0.295° ± 0.369° (33 ± 41 km), and longitudinal error averaged 0.185° ± 0.392° (21 ± 44 km).

### 2.3 Genetic Variable Calculations

We developed microsatellite primers using nuclear DNA extracted from blood samples collected from the 2019 population (McGee et al. 2025). We used eight species‐specific primers for genetic analysis (Table 2) in a Principal Components Analysis performed on individual microsatellite loci using tables of allele proportions, or frequencies (%PCA), followed by a multiple co‐inertia analysis (MCOA), both run with the *adegenet* package in R (Jombart 2008). Unlike a traditional Principal Components Analysis (PCA), which combines all loci in a single analysis from the outset, a paired %PCA and MCOA analysis summarizes the genetic variation captured by individual loci, reducing the dimensionality of each locus to just a few orthogonal axes, so that all loci can then be compared on a common basis using MCOA, and a consensus geometric structure sought (Putman and Carbone 2014). A %PCA produces the same axes as a classic PCA; however, instead of centering the data, points are placed by averaging with respect to allele frequencies (Laloë et al. 2007). MCOA is an ordination technique that combines the principal components produced by multiple loci, accounting for variance in alleles across all loci, and preventing loci with many alleles or those under unique forms of selection from dominating (Bady et al. 2004; Laloë et al. 2007). All missing allele values (2.24%) were replaced with the most common allele at each locus (Särkkä et al. 2025). We cross‐checked all birds in this study to historic nesting data to ensure there were no known genetic or social nesting relationships between individuals. The first three axes (henceforth referred to as Composite Genotype Score 1, 2, or 3) consolidated by the MCOA summarized 30% of the genetic variation in the population, thus creating three composite genetic variables with which to describe genetic relatedness among members of the same population (Table 3). Individuals with more similar scores within each of these three variables were presumed to be more closely related.

**TABLE 2:** Summary of primers used to calculate genetic variables (*N* = number of genotypes successfully scored for the characterization of genetic variation, *H*_*o*_ = observed heterozygosity, *H*_*e*_ *=* expected heterozygosity, and *F*_*IS*_ = inbreeding coefficient).

SSR locus	*N*	Number of alleles observed	Effective number of alleles	*H*_*o*_	*H*_*e*_	*F*_*IS*_
BOBO‐01	152	7	3.289	0.664	0.696	0.045
BOBO‐02	147	9	4.595	0.803	0.782	−0.026
BOBO‐03	152	11	4.550	0.743	0.780	0.047
BOBO‐04	138	13	4.209	0.819	0.762	−0.074
BOBO‐05	150	18	8.738	0.847	0.886	0.044
BOBO‐06	139	14	5.780	0.842	0.827	−0.018
BOBO‐08	147	10	6.361	0.844	0.843	−0.001
BOBO‐09	133	12	6.996	0.842	0.857	0.017

**TABLE 3:** Percent of genetic variation accounted for in the Genetic Variables 1, 2, 3.

Variable	Eigenvalue	Proportion of total genetic variation
Genetic Variable 1	3.042	0.11
Genetic Variable 2	2.736	0.10
Genetic Variable 3	2.525	0.09

### 2.4 Data Preparation and Statistics

Bobolinks are frequently described as wanderers, with our population exhibiting highly variable movement (latitude and longitude) as they forage in preparation for both fall and spring migration (Renfrew et al. 2019; McGee, unpublished). For the purposes of this study, we characterized the start of migration to be consistent movement in either a southern or northern direction for fall and spring migration, respectively. Prior to the start date, individuals were either stationary or moving erratically. We also highlighted the timing and location of the major water crossings prior to arriving and departing South America, due to the risky nature of prolonged overwater flights for passerines. While full migration studies are complex, we attempted to distill the Bobolink's multi‐continent movement data into key events.

**FIGURE 1:**
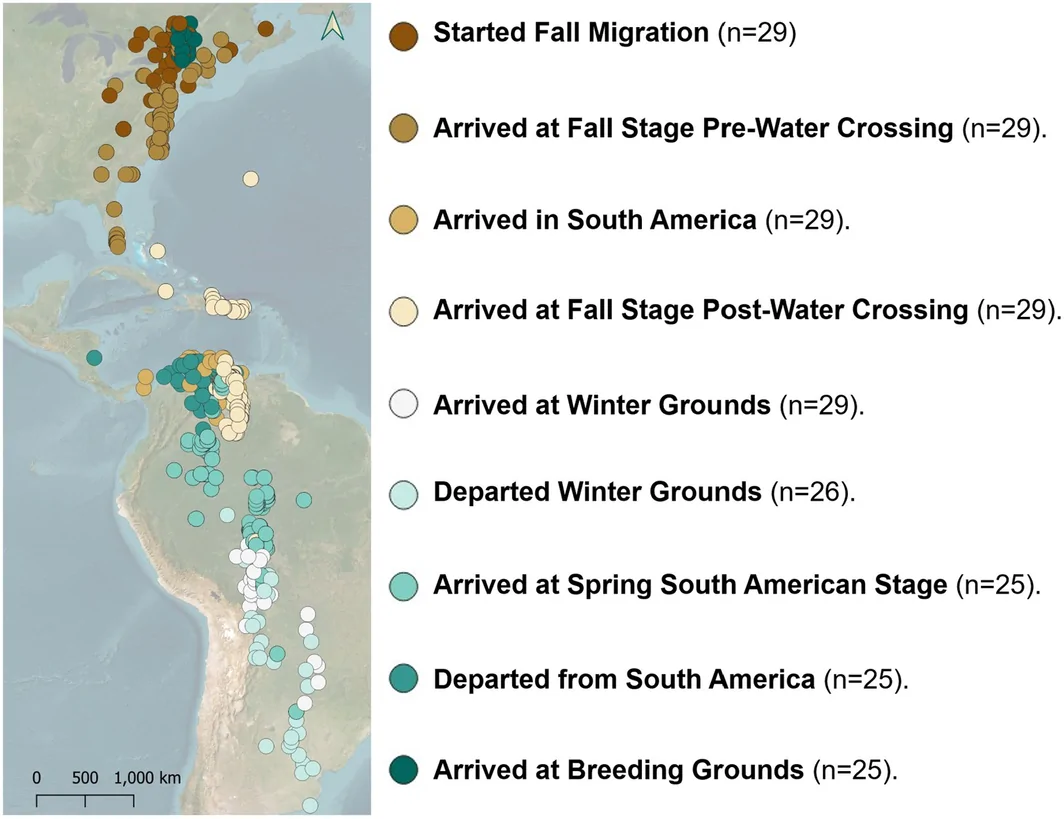
The full annual migration cycle of 29 Bobolinks equipped with geolocators, plotted as nine distinct migration events, seven of which were the focus of this study. Two South American stages, fall post‐water crossing and spring South American stage, were not independently analyzed but modeled as carryover effects within the analysis of other migration events.

We identified nine distinct migration events across the Bobolink's annual cycle (Figure 1) from which to explore spatial and temporal variation in migration behavior. (1) Started Fall Migration: defined by the first day of consistent southern movement (< 5 northern movements detected) after which the bird only flew south (*n* = 29). (2) Arrived at Fall Stage, Pre‐Water Crossing: defined by the longest stopover each bird made before its first water crossing (*n* = 29). (3) Arrived in South America: defined by the first point where each bird was detected in continental South America (south latitude 12 N) (*n* = 29). (4) Arrived at Fall Stage, Post‐Water Crossing: defined by the longest stop each bird made after completing its first water crossing of fall migration (*n* = 29). (5) Arrived at Winter Grounds: defined by the first long pause (> 7 days) in long‐distance (> 100 km) southern flights located south of the equator (*n* = 29). (6) Departed Winter Grounds: defined by the last day each bird spent within its wintering ground before it started consistent northern movement (< 5 southern movements detected) for spring migration (*n* = 26). (7) Arrived at Spring South American Stage: defined by the longest stopover each bird took in South America on its spring migration (*n* = 25). (8) Departed from South America: defined by the location where each bird departed on its first spring water‐crossing, leaving South America (*n* = 25). (9) Arrived at Breeding Grounds: defined by the day each bird ended northern movement during spring migration and arrived at its breeding grounds (*n* = 25).

Seven of these nine migration events were the focus of this study, with two South American stages (fall, post water‐crossing and South American spring stage) used only as possible carryover effects in the analysis of other migration events. Thus, our analyses focused exclusively on major timing events such as the beginning and end of fall and spring migrations, as well as staging areas for large water crossings. New movements were considered to begin when a bird crossed the geolocator error radius (1.2° latitude, 0.5° longitude, adapted from Renfrew et al. 2013) around the location of each event. We categorized stopovers as any location a bird utilized for at least 24 h without leaving the error radius (Warnock 2010). We converted dates into “Day of Year” with values ranging between 1 and 365. To analyze climate, we utilized historic climate reanalysis data daily averages for temperature (°C) (Marra et al. 2005; Saino et al. 2007), total precipitation (mm) (Brlík et al. 2022; Rüppel et al. 2023), and mean sea level pressure (Pa) (Rüppel et al. 2023) from the European Centre for Medium‐Range Weather Forecast (ECMWF) ERA5 Hourly Averages (0.5° × 0.5° resolution). Hourly data were averaged for a 24‐h floating window around the time a geolocator point was collected to create daily averages at each point. We used QGIS 3.28.3 to map each bird's migration route from FLightR.

For each of the seven migration events, both timing (Day of Year, hereafter DoY) and location (latitude) were treated as separate response variables for assessing correlated genetic effects (composite genotype score 1, 2, 3) and carryover effects from the previous migration events (see Appendix S1 for full model details). We included carryover effects such as timing and location of departure from the preceding event and the start of the seasonal migration. We also measured carryover effects of average environmental conditions (temperature, mean sea level pressure (MSL), and total precipitation (TP)) before the departure from the site of the previous event. In addition, each multiple linear regression model included sex as a factor and its interaction with all other variables, except genetic variables. To determine if year was significantly impacting timing or location, we conducted a one‐way ANOVA prior to running each migration event model. If year was significant, it was then included in the full model. We ran all multiple linear regressions in R version 4.3.2 followed by a process of backward model selection based on Akaike's Information Criterion (AIC; Appendix S1), and present ANOVA results only for best fitting models describing each event (Tables S1–S10). Partial *R* ^2^ values calculated with the R package *domir* were included in table results to show the contribution each variable had towards the optimal model's overall *R* ^2^ value (Luchman 2021).

## 3 Results

### 3.1 Started Fall Migration

**FIGURE 2:**
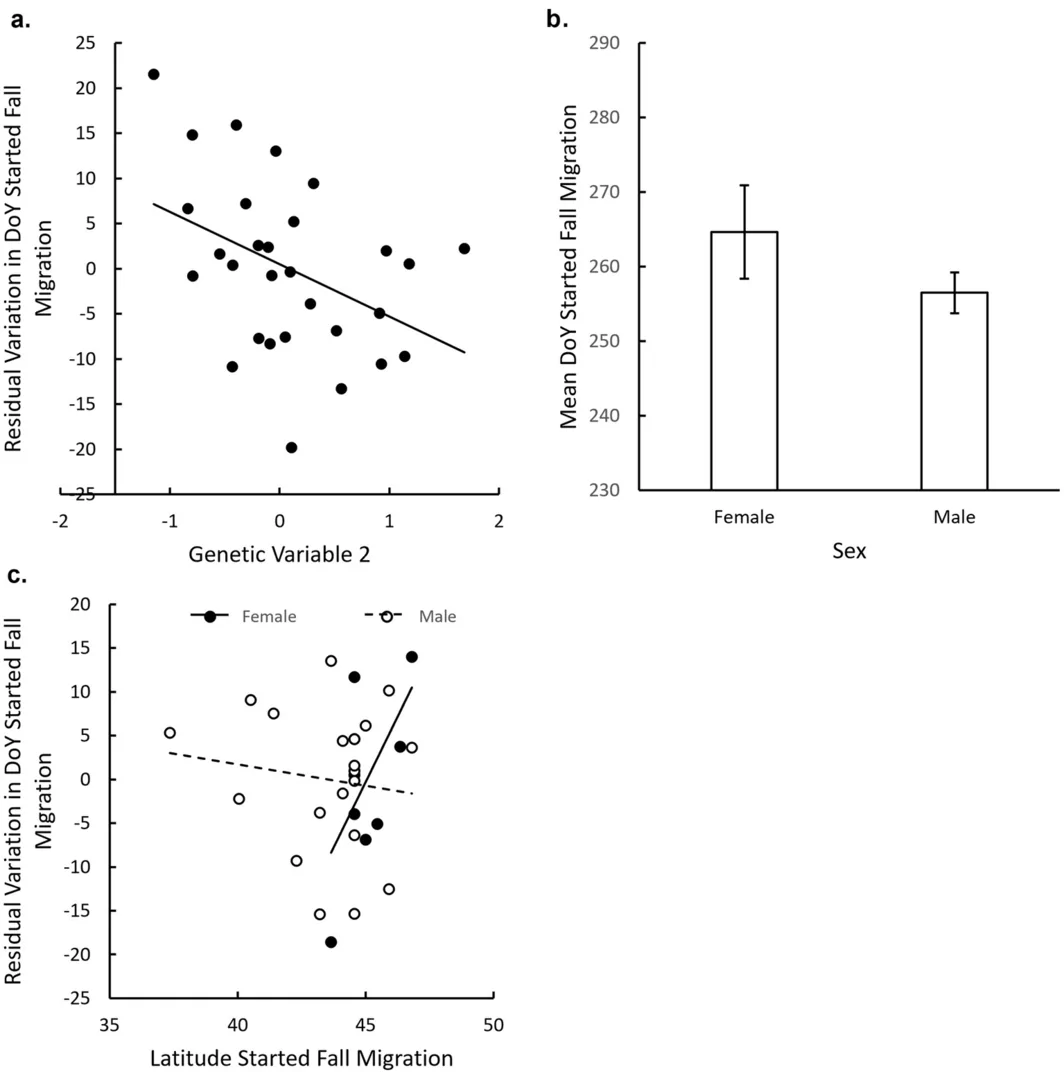
The start of Bobolink's fall migration was correlated temporally with genetics (a), sex (b), and latitude (c). Residuals are the leftover variation in the response variable after the variation explained by all other explanatory variables besides the explanatory variable of interest has been factored out.

The timing of fall migration, defined by the day of first consistent southern movement, was influenced by the composite genotype score (partial *R* ^2^ = 0.224) and sex‐specific differences in latitude (partial *R* ^2^ = 0.139). Overall, the start of fall migration date showed a significant effect of composite genotype score (Figure 2a, Table S1), sex (Figure 2b), and the interaction between sex and latitude (Figure 2c). Males ($\bar{x}$ = day 256.5, SD = 12.3) started fall migration earlier than females ($\bar{x}$ = day 264.7, SD = 18.8), and left earlier from higher latitudes than females; females were more constrained in the range of latitudes they departed from.

**FIGURE 3:**
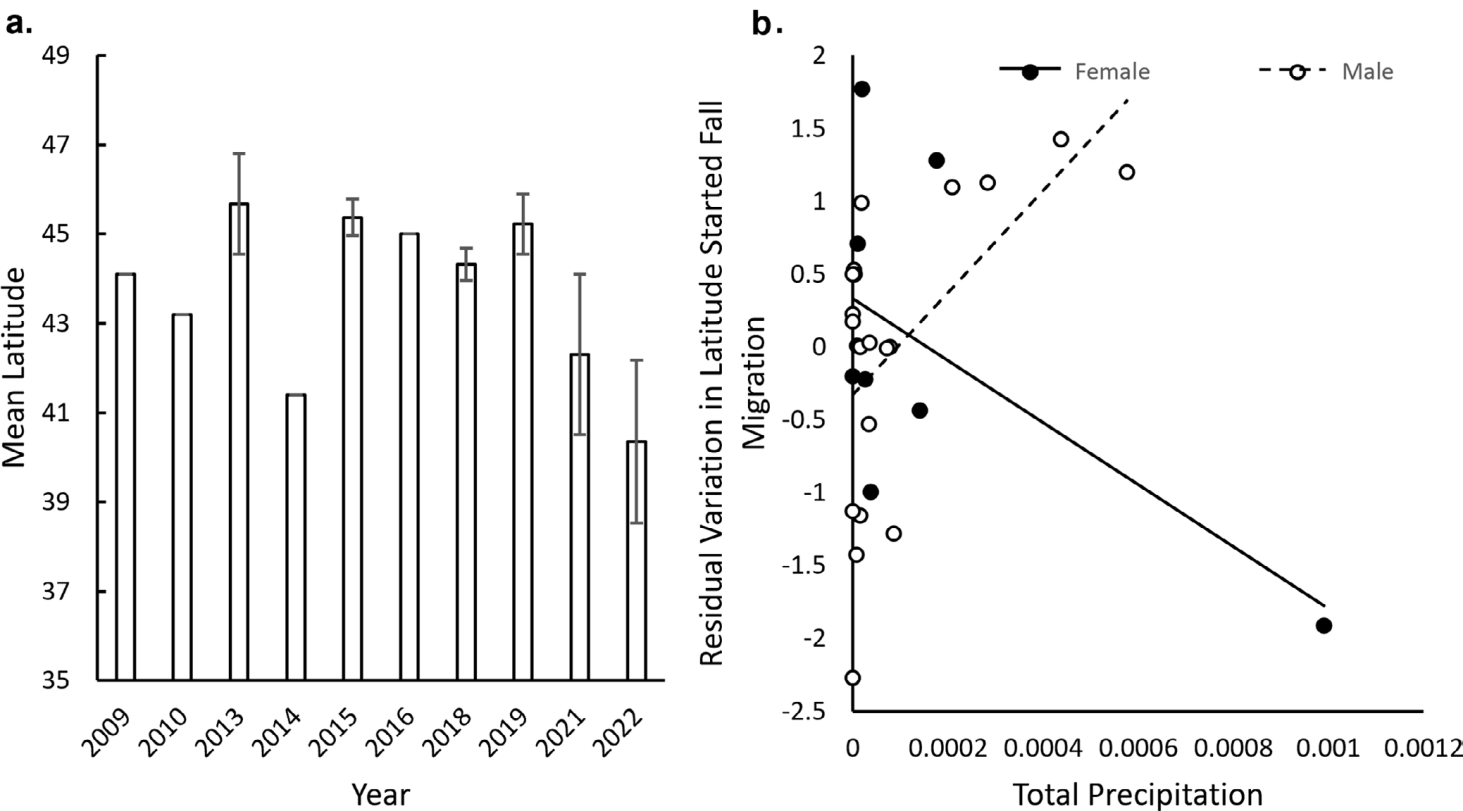
The start of fall migration varied latitudinally with (a) year (error bars depict standard error), and the interaction between sex and (b) total precipitation. Residuals are the leftover variation in the response variable after the variation explained by all other explanatory variables besides the explanatory variable of interest has been factored out.

The mean latitude from which Bobolinks started their fall migration varied year to year from 45.68° N (±1.12 SE) to 40.35° N (±1.82 SE), which contributed both a relatively high partial *R* ^2^ (0.517) and significance in the ANOVA model (Figure 3a, Table S1). The only other variable contributing significantly to the starting latitude of fall migration was total precipitation, which interacted significantly with sex (Figure 3b). Precipitation across the range of latitudes from which birds began their fall migration was generally very low. However, one female starting her migration from an extremely southern latitude experienced an unusually high amount of rainfall, creating an apparent trend of higher precipitation pushing females out of more southerly latitudes. Males experienced more precipitation when they left from northern latitudes.

### 3.2 Fall Stage, Pre‐Water Crossing

**FIGURE 4:**
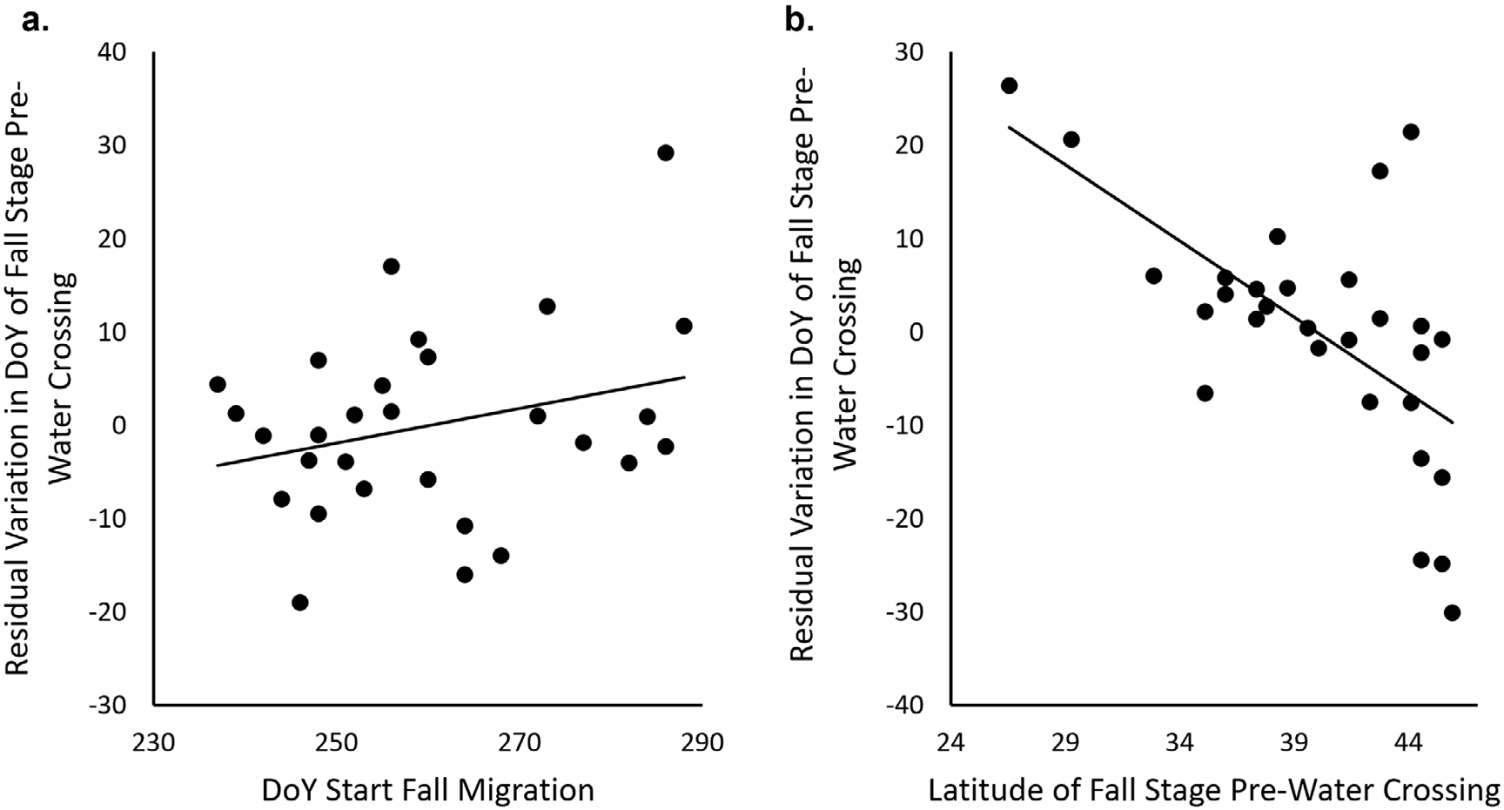
The timing of fall stage, pre‐water crossing was correlated with carry over effects from the start of fall migration (a) and latitude (b). Residuals are the leftover variation in the response variable after the variation explained by all other explanatory variables besides the explanatory variable of interest has been factored out.

The fall stage, pre‐water crossing, was the longest duration stopover birds utilized in North America during fall migration. The timing of the stage was significantly impacted by carry‐over effects from the start date of fall migration (Figure 4a, Table S2). In both sexes, birds arrived at their stage earlier if they started fall migration earlier (partial *R* ^2^ = 0.173). Seven females and two males staged at the breeding grounds and therefore had earlier staging dates than migration start dates. Latitude also played a significant role in the DoY model (Figure 4b), with birds staging farther north arriving earlier to their staging grounds.

**FIGURE 5:**
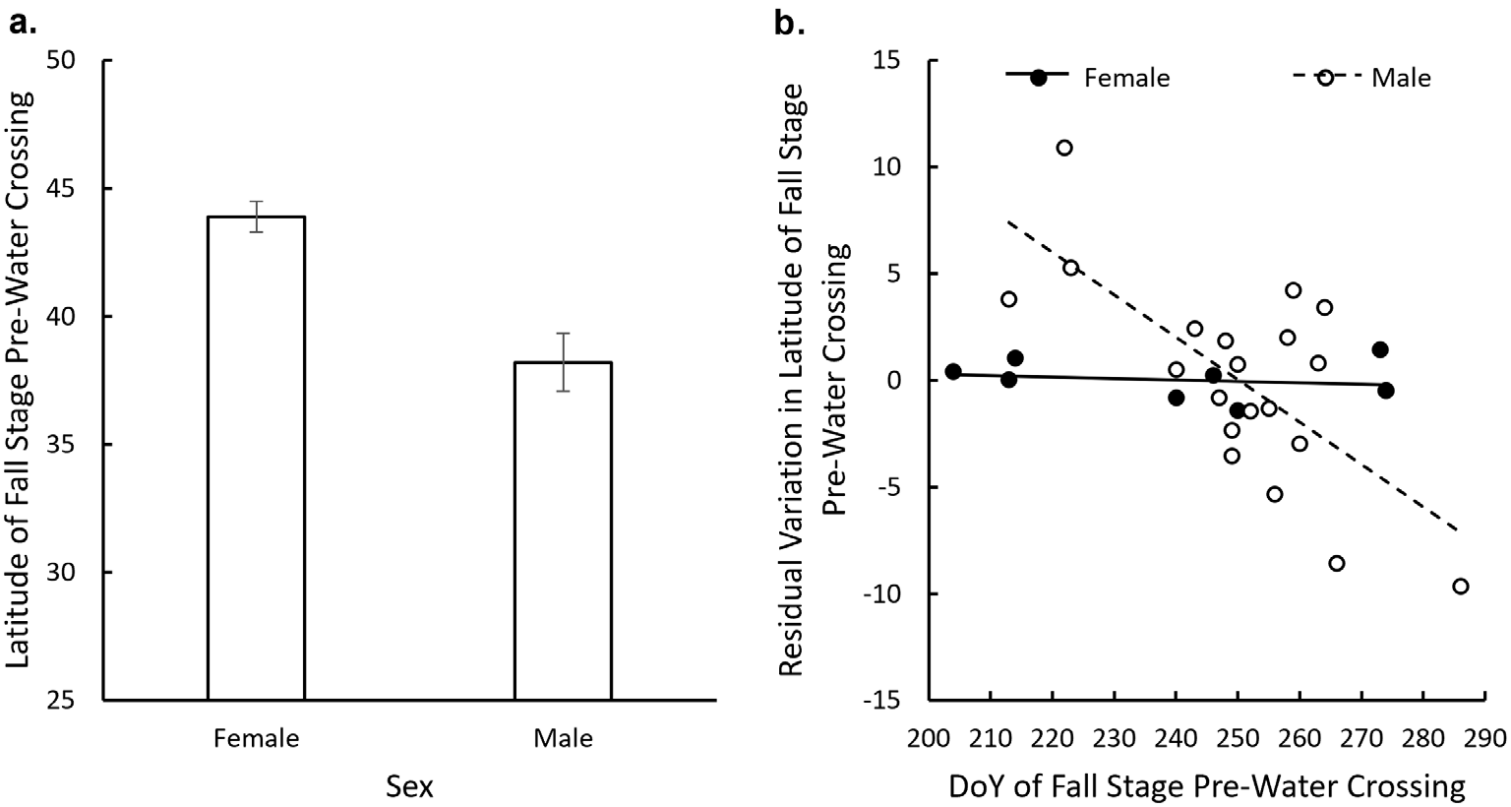
The location of fall stage, pre‐water crossing was correlated with sex (a) and DoY of fall stage pre‐water crossing (b). Error bars depict standard deviation. Residuals are the leftover variation in the response variable after the variation explained by all other explanatory variables besides the explanatory variable of interest has been factored out.

The latitude of the fall stage, pre‐water crossing, was significantly correlated with sex (Figure 5a, Table S2), with females staging farther north on average ($\bar{x}$ = 43.9, SD = 1.699) and over a smaller range of latitudes than males ($\bar{x}$ = 38.205, SD = 4.944). Sex also interacted with DoY in this model of fall stage latitude, with arrival date having little effect on female staging latitude, whereas earlier arriving males staged farther north than later arriving males (Figure 5b). The partial *R* ^2^ for the interaction between sex and DoY accounted for half of the total variation explained by this latitude model (0.417).

### 3.3 Arrival to South America

**FIGURE 6:**
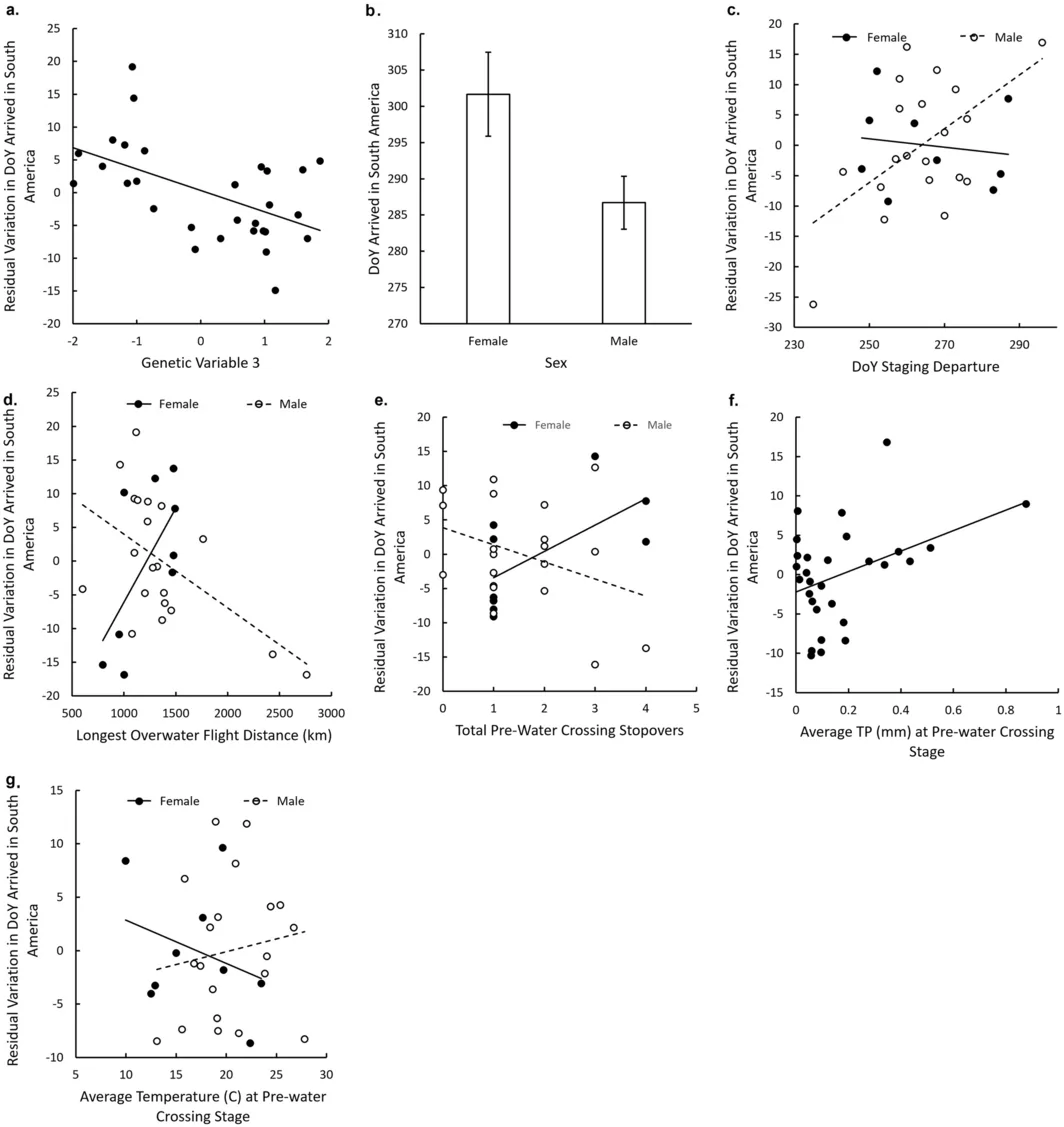
The timing of Bobolink arrival in South America was correlated with genetics (a), sex (b), and carry over effects from timing of departure from the fall pre‐water crossing stage (c), longest over water flight distance (d), and total pre‐water crossing stopovers (e). Weather variables total precipitation (TP) (f) and temperature (g) were both significant but followed seasonal weather patterns. Error bars depict standard deviation. Residuals are the leftover variation in the response variable after the variation explained by all other explanatory variables besides the explanatory variable of interest has been factored out.

Timing of arrival to South America was correlated with Genetic Variable 3 (Figure 6a, Table S3), albeit weakly (*R* ^2^ = 0.053). Females arrived in South America significantly later than males (Figure 6b, Table S3), but only males were influenced by carry over effects of timing departure from pre‐water crossing staging grounds (Figure 6c). Based on the interaction with sex, males that left their staging area earlier arrived in South America earlier. There was also a significant interaction (Figure 6d) and relatively high partial *R* ^2^ (0.155) attributed to sex and longest overwater flight distance. The majority of birds took 1000–1500 km flights, but within that range there was a positive correlation between longer flights and later arrival dates in females, which was paradoxically reversed in males. Similarly, while females making more pre‐water crossing stopovers arrived later to South America, the opposite trend was apparent in males (Figure 6e). Males departing later from fall staging areas increased pressure to fly directly to South America. Most individuals experienced little rain at their staging areas (all birds < 1.0 mm; Figure 6f), but two birds experiencing above‐average rainfall (~0.5, 0.8 mm) arrived in South America relatively late, which produced a significant positive correlation with precipitation (Figure 2f). Temperature contributed a low but significant partial *R* ^2^ (0.05) to the model, with the significance of the interaction (Figure 6g) with sex driven by opposite trends between males and females. Females arrived later to South America, driven by colder temperatures in North America, whereas males arrived earlier from colder temperatures, reflecting shorter‐term weather events impacting their relatively low latitude staging areas (Figure 6g). Arrival latitude in South America was not significantly impacted by any variables in our model set.

### 3.4 Arrival to Winter Grounds

**FIGURE 7:**
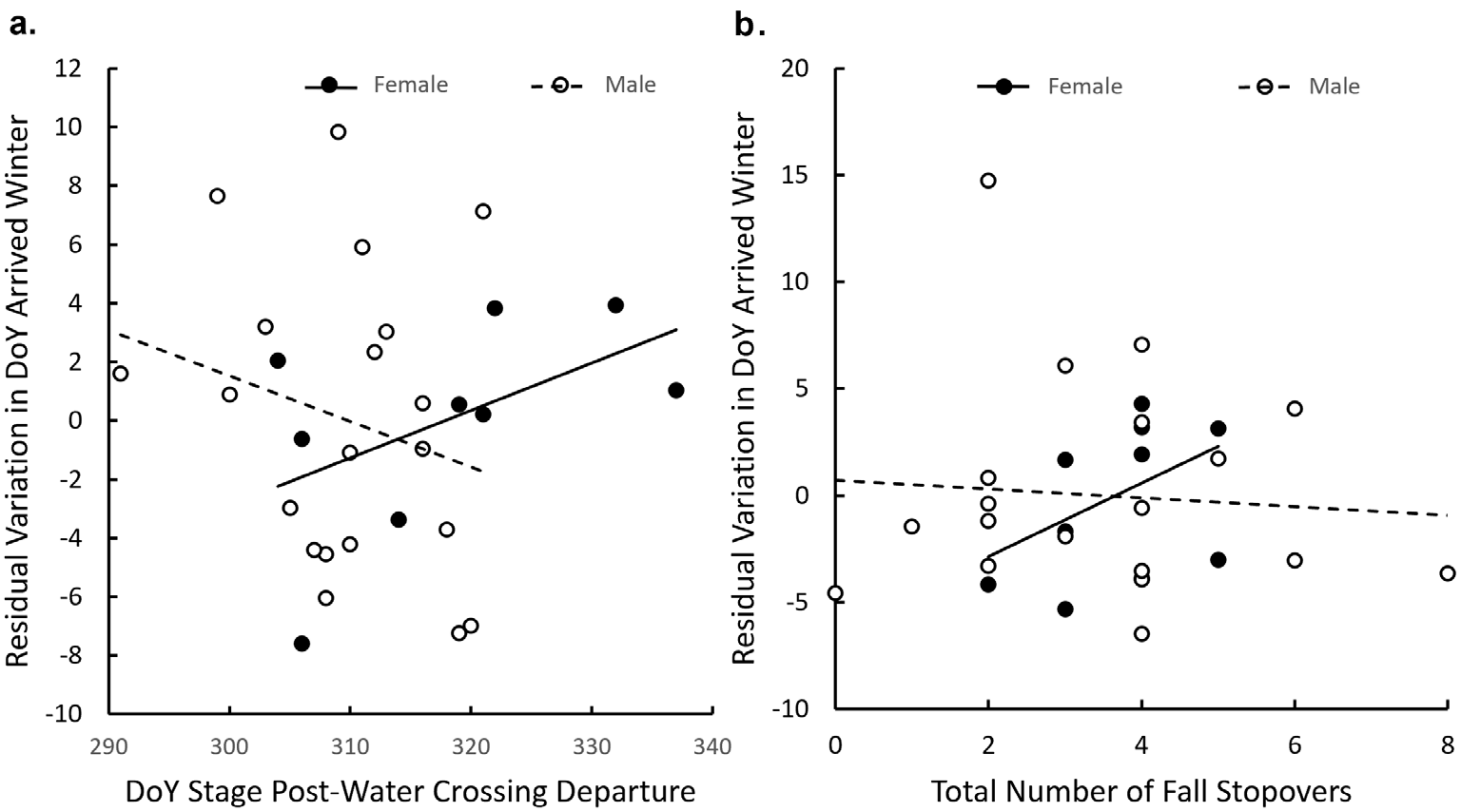
Bobolink arrival at their first wintering grounds marked the end of the fall migration. Temporally, wintering ground arrival saw carry over effects from stage departure (a) and the total number of fall stopovers (b). Residuals are the leftover variation in the response variable after the variation explained by all other explanatory variables besides the explanatory variable of interest has been factored out.

Arrival at the wintering grounds, when the bird stopped making consistent southern movements, showed stronger carry over effects for females than males. Females that left their South American fall staging grounds earlier arrived at their wintering grounds ($\bar{x}$ = day 333.1, SD = 10.9) earlier, whereas males exhibited the opposite trend (Figure 7a, Table S4). Males showed little variation in wintering ground arrival date relative to the number of stopovers they made along the way, which at least in part resulted from a narrow range of arrival dates ($\bar{x}$ = day 322.5, SD = 6.1), whereas females with greater numbers of stopovers arrived later. This interaction contributed the largest partial *R* ^2^ to the DoY model (0.209). Hours spent at post‐water crossing stage (Table S4) had a positive correlation with arrival timing, although the effect was very weak (*R* ^2^ = 0.025).

**FIGURE 8:**
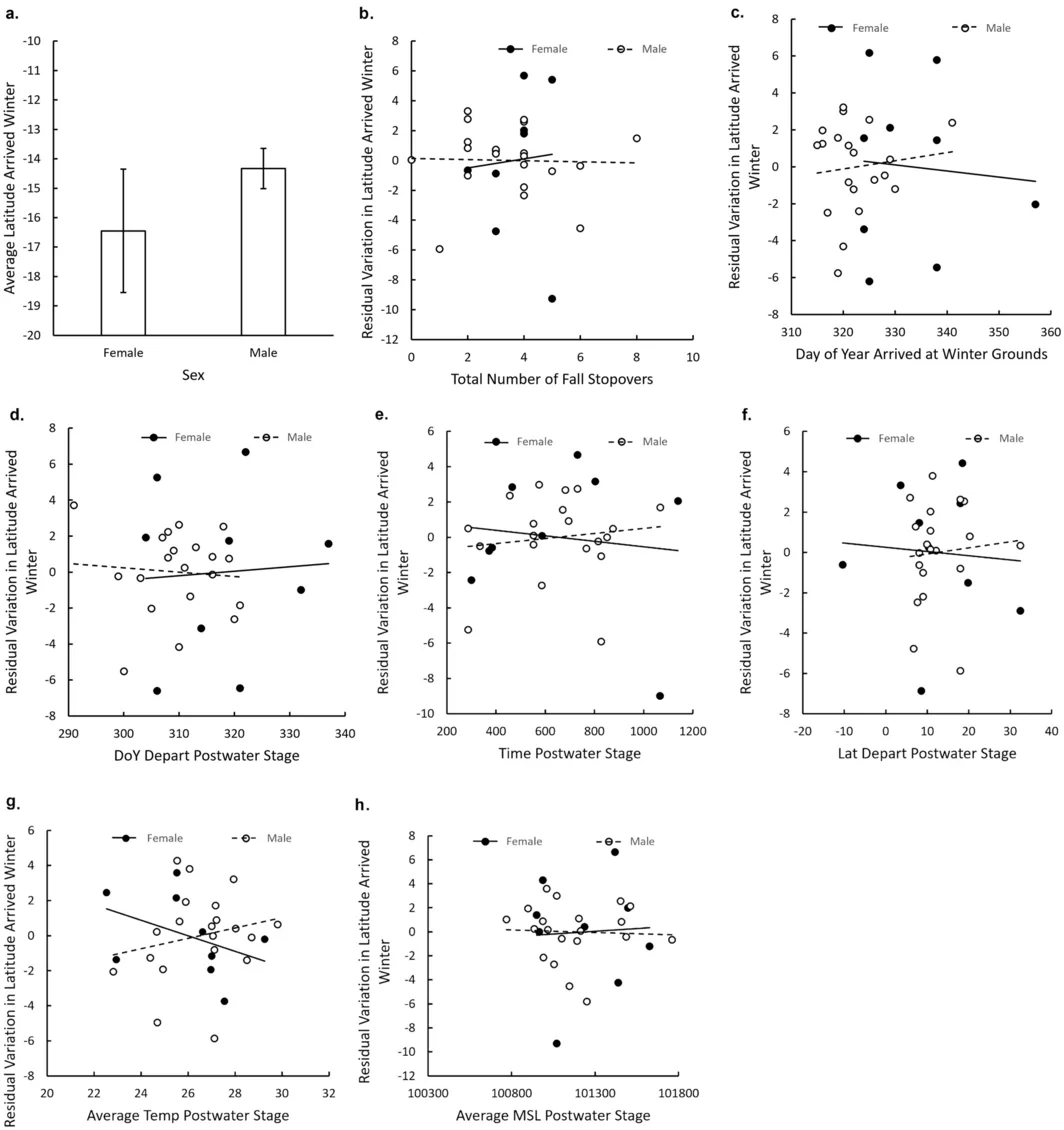
Bobolink arrival at their first wintering grounds marked the end of the fall migration. Spatially, wintering arrival location was correlated with sex (a), total number of fall stopovers (b), DoY arrived at winter grounds (c), DoY departed post‐water stage (d), time spent at post‐water stage (e), latitude of the post‐water crossing fall stage (f), average post‐water stage temperature (g), and average barometric pressure at the post‐water stage (h). Error bars depict standard deviation. Residuals are the leftover variation in the response variable after the variation explained by all other explanatory variables besides the explanatory variable of interest has been factored out.

Wintering grounds latitude differed notably between sexes, with males wintering farther north and over a smaller range than females (Figure 8a, Table S4). Additional factors explaining the latitude of winter arrival were numerous and complex and included the total number of fall stopovers, the timing of winter arrival, and a host of carryover effects related to the post‐water staging area, all interacting with sex (Table S4). Females were constrained to 2–4 stopovers and tended to settle at higher winter latitudes following more numerous stopovers, whereas the number of stopovers was more variable in males and had little impact on winter ground latitude (Figure 8b). Females arrived later to their wintering grounds than males, and the later they arrived the farther south they settled, whereas late arriving males tended to settle farther north (Figure 8c). Carryover effects from the post‐water stage on winter ground latitude included (1) the timing of departure from the post‐water stopover, with females leaving later tending to settle farther north, whereas males leaving later settled farther south (Figure 8d); (2) the time spent at the post‐water stopover, with females who remained longer tending to settle farther south for the winter, whereas males remaining longer settled farther north (Figure 8e); (3) the latitude of the post‐water stopover, with females stopping at more northerly sites settling farther south for the winter, whereas male post‐water stopover latitude and winter ground latitude were positively correlated (Figure 8f); (4) the average temperature at the post‐water stopover, with females experiencing relatively lower temperatures wintering farther north, whereas males experiencing colder temperatures settled farther south (Figure 8g); and (5) average barometric pressure at the post‐water stopover, with females experiencing low pressure settling slightly farther south for the winter, whereas males were little affected by pressure (Figure 8h).

### 3.5 Depart Wintering Grounds

**FIGURE 9:**
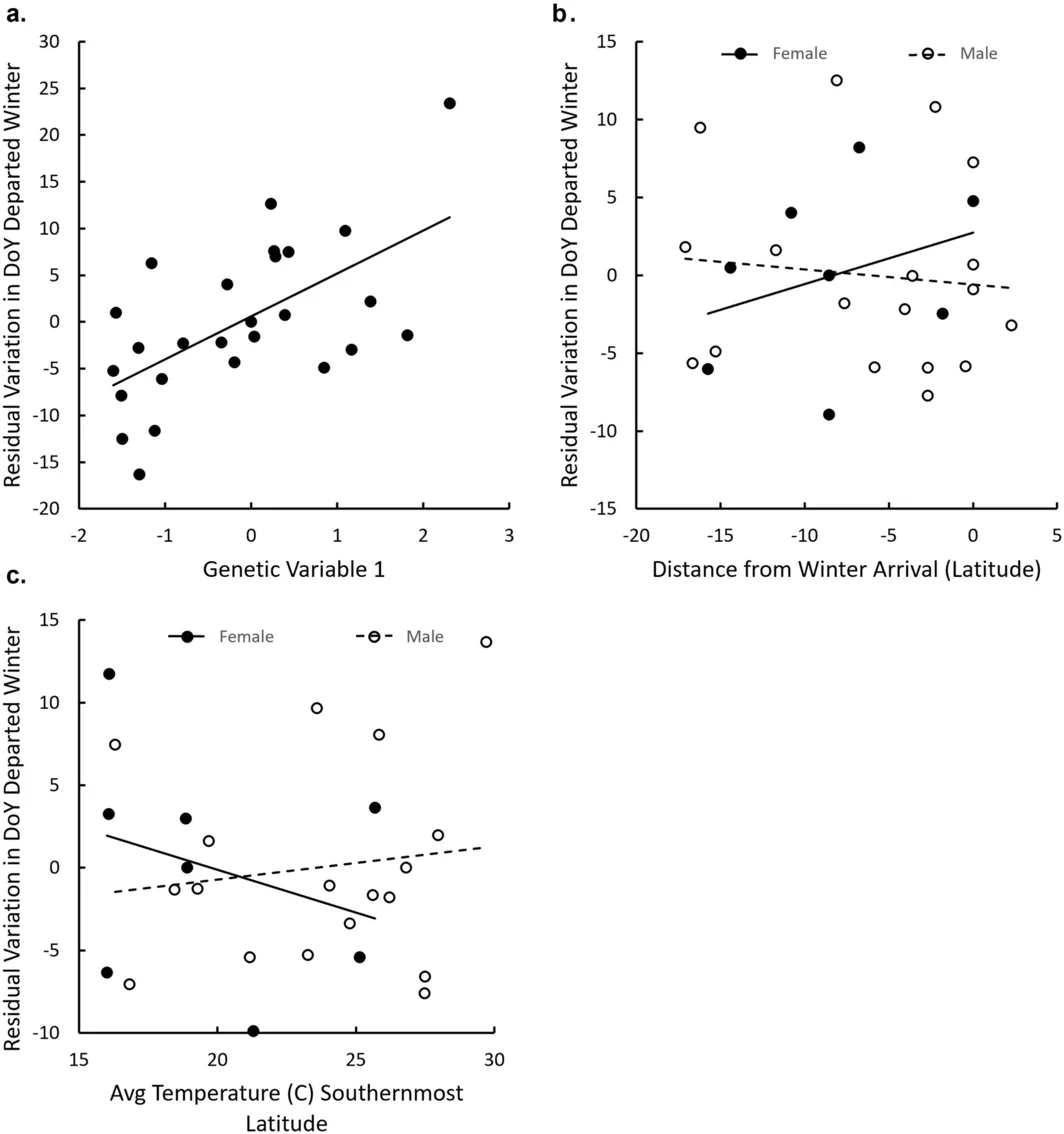
Bobolinks were nomadic during their non‐breeding season and the departure from their last wintering grounds marked the start of spring migration. Timing was correlated with genetics (a), weakly with distance from winter arrival (b), and with environmental effects of temperature (c). Residuals are the leftover variation in the response variable after the variation explained by all other explanatory variables besides the explanatory variable of interest has been factored out.

Nearly 81% of Bobolinks occupied more than one winter stopover, as defined by movement to new locations outside of the geolocator error radius. We defined the departure from wintering grounds as the first date of consistent northern movement, and therefore the start of spring migration. 88.5% of birds departed their wintering grounds from their southernmost winter stopover (Table S5). The timing of winter departure was significantly correlated with composite genotype score 1 (Figure 9a, Table S5), with more genetically similar birds leaving at similar dates. There was also a weak correlation between winter departure and the distance a bird's final winter stopover was from its winter arrival location, which varied by sex (Figure 9b). Females that traveled the farthest south during their winter movements departed earliest, whereas male departure was not impacted by their winter movements. The interactive effect of temperature and sex on winter departure date (partial *R* ^2^ = 0.055), on the other hand, showed opposite impacts of temperature on male and female departure, with females leaving later with cooler weather and males leaving earlier (Figure 9c).

**FIGURE 10:**
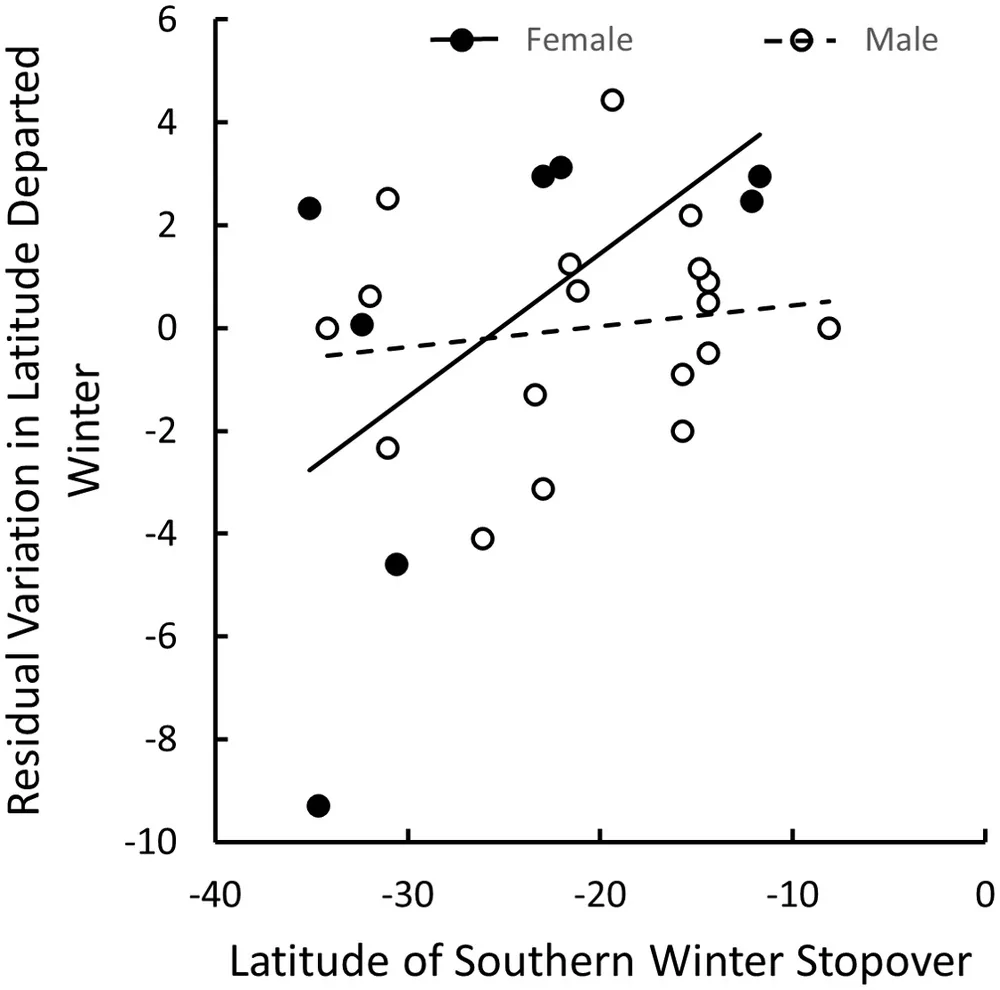
Departure from the Bobolinks' wintering grounds marked the start of spring migration. Location of departure was impacted by carry over effects from the winter arrival location. Residuals are the leftover variation in the response variable after the variation explained by all other explanatory variables besides the explanatory variable of interest has been factored out.

All birds in the study started spring migration from their southernmost latitude; however, 11.5% of birds made additional southern movements past their southern‐most winter stopover before starting northern migration (Table S5). In spite of this behavior, there was still a positive correlation between the latitude of the final winter stopover and the latitude of departure from South America for both sexes, although this correlation was more pronounced for females (Figure 10).

### 3.6 Departure From South America

**FIGURE 11:**
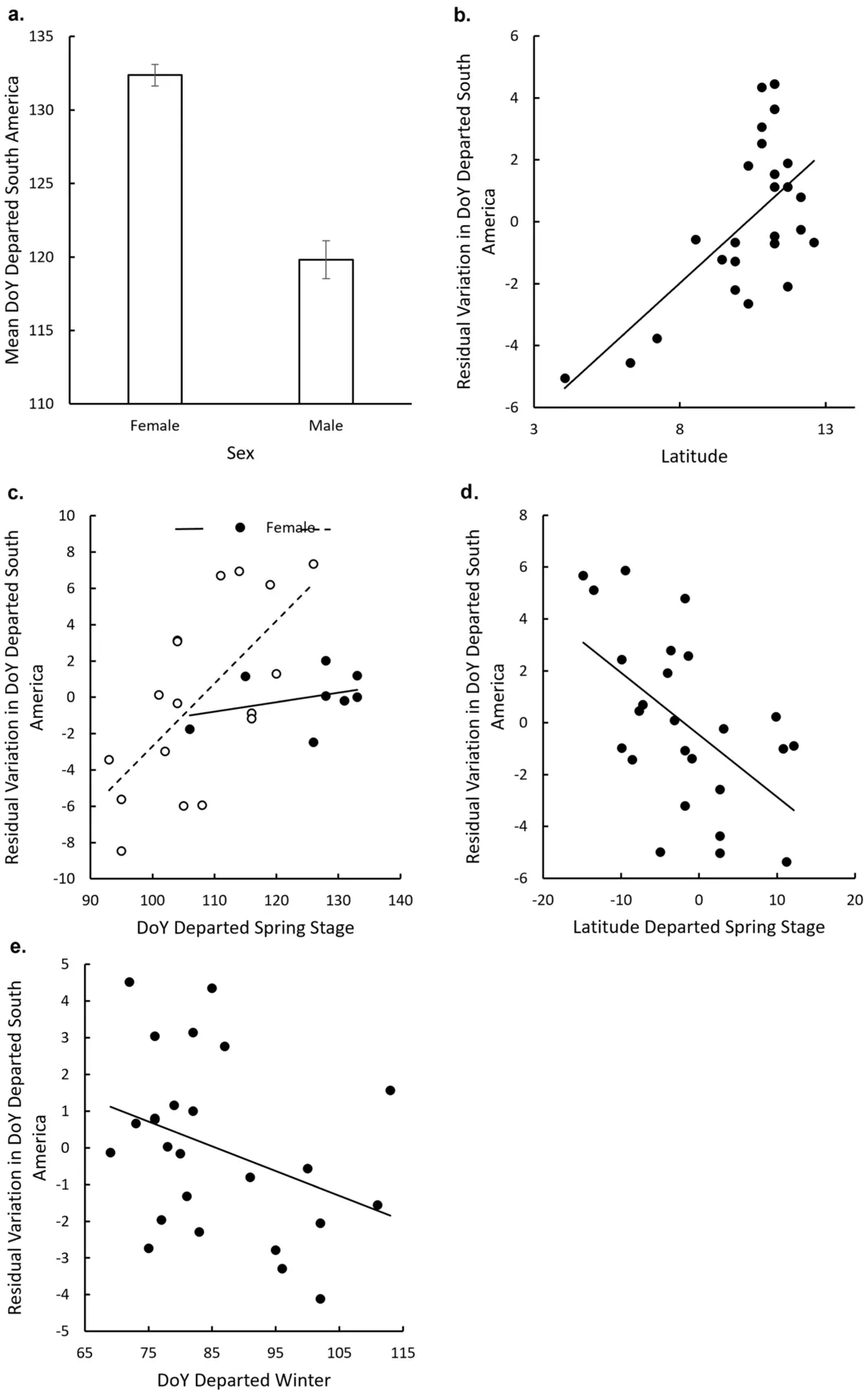
South American departure timing was correlated with sex (a), latitude (b), and carry over effects from spring stage departure date (c) and latitude (d), and winter departure date (e). Error bars depict standard deviation. Residuals are the leftover variation in the response variable after the variation explained by all other explanatory variables besides the explanatory variable of interest has been factored out.

Departure from South America brought more pronounced timing differences between the sexes (Figure 11a, Table S6). Males ($\bar{x}$ = day 119.82, SD = 5.3) left South America significantly earlier than females ($\bar{x}$ = day 132.38, SD = 2.0). In both sexes, birds that departed from farther south departed South America earlier (Figure 11b). The interaction between sex and spring stage departure (partial *R* ^2^ = 0.248) showed males that left their staging grounds earlier left South America earlier, whereas females showed less variation in the dates on which they left South America (Figure 11c, Table S6). Other carryover effects impacting both sexes equally were apparent as birds with more southerly staging areas leaving South America later (Figure 11d), and birds who left their wintering grounds earlier lingered in South America longer (Figure 11e).

**FIGURE 12:**
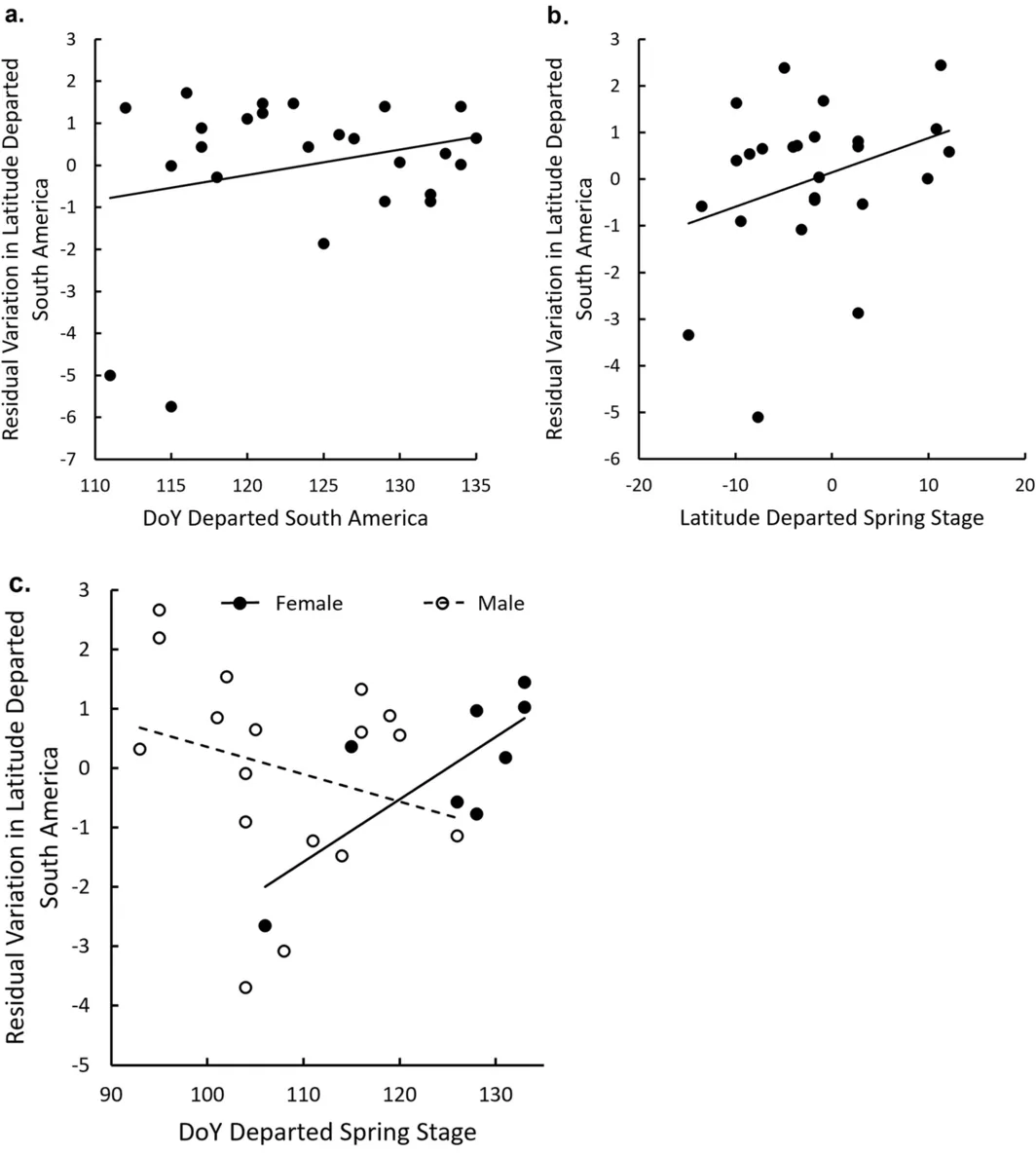
South American departure latitude was influenced by South American departure DOY (a) and carry over effects from spring stage departure latitude (b) and DoY (c). Residuals are the leftover variation in the response variable after the variation explained by all other explanatory variables besides the explanatory variable of interest has been factored out.

There was a significant (Figure 12a, Table S6) positive correlation (partial *R* ^2^ = 0.188) between latitude and departure date, where birds that started their South American departure flight from farther south departed earlier. Spring stage impacted the latitude of South American departure through both its positive latitudinal effect (Figure 12b) and its timing, although the latter effect was sex‐dependent (Figure 12c). Females leaving their spring staging areas early left South America from more southerly latitudes, whereas males leaving their staging areas early left South America from farther north. The model identified a significant difference between sexes; however, the correlation was weak (partial *R* ^2^ = 0.045) and the difference between the average latitudes of both sexes was within the error range of the geolocators (male $\bar{x}$ = 10.27 N, female $\bar{x}$ = 10.40 N).

### 3.7 Breeding Ground Arrival

**FIGURE 13:**
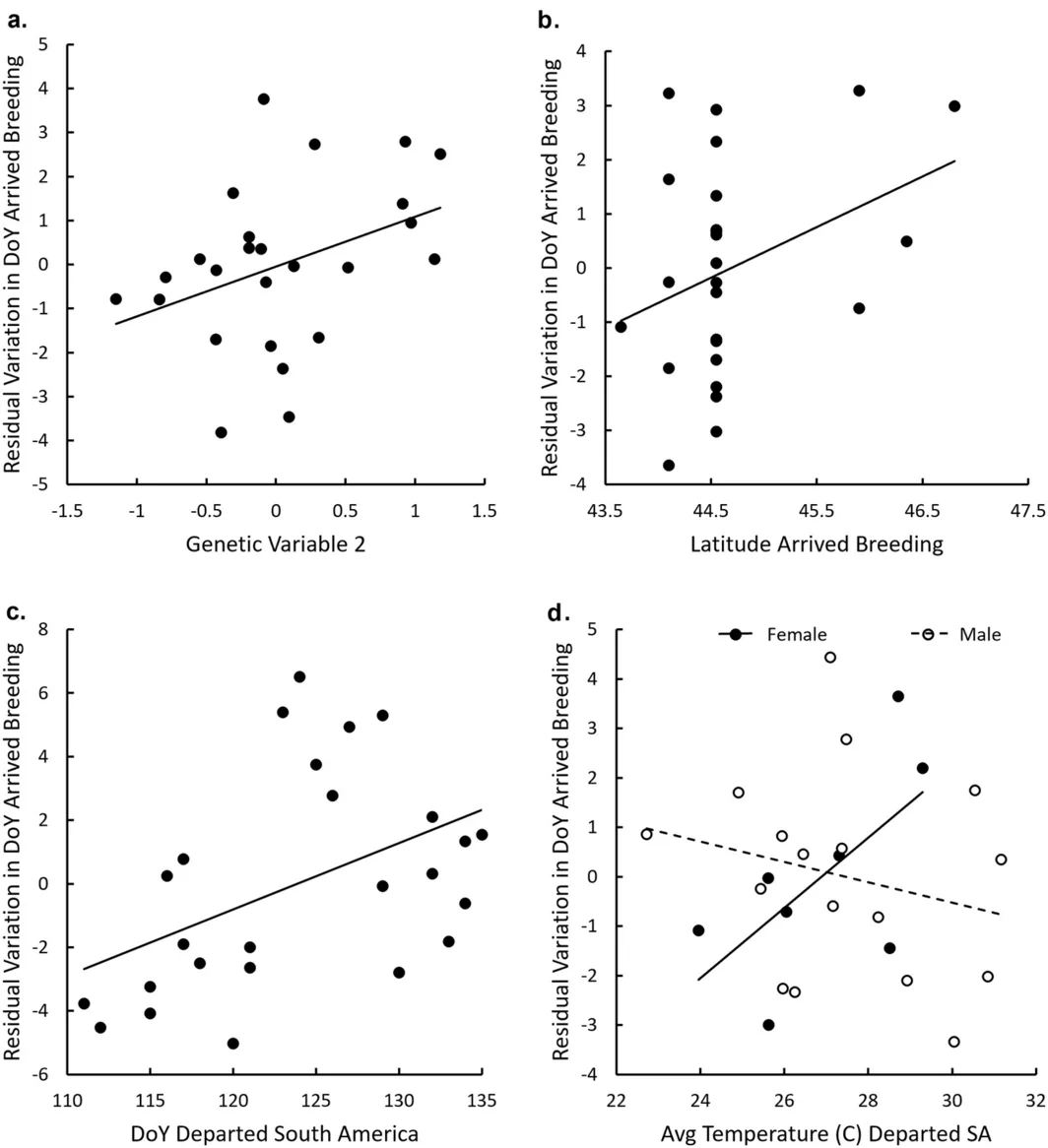
The timing of Bobolink breeding ground arrival was correlated with Genetic Variable 2 (a) latitude (b), carryover effects from departure from South America (c), and an interaction between sex and temperature (d). Residuals are the leftover variation in the response variable after the variation explained by all other explanatory variables besides the explanatory variable of interest has been factored out.

The timing of breeding ground arrival was significantly related to composite genotype score 2, albeit weakly (*R* ^2^ = 0.016, Figure 13a, Table S7), and latitude of the breeding ground (Figure 13b; Table S7). Individuals breeding at higher latitudes arrived slightly later than those breeding at lower latitudes. Breeding ground arrival was also heavily influenced by a carryover effect from date of departure from South America (*R* ^2^ = 0.322, Figure 13c). The model did not identify a significant difference between arrival timing between sexes (Table S7) despite males arriving earlier on average (male $\bar{x}$ = day 134.29, SD = 4.8; female $\bar{x}$ = day 145.0, SD = 4.3). The interaction between temperature at South American departure and sex had a significant effect on breeding ground arrival, where females arrived later than males and later arrivals correlated to warmer spring weather only in females (Figure 13d).

**FIGURE 14:**
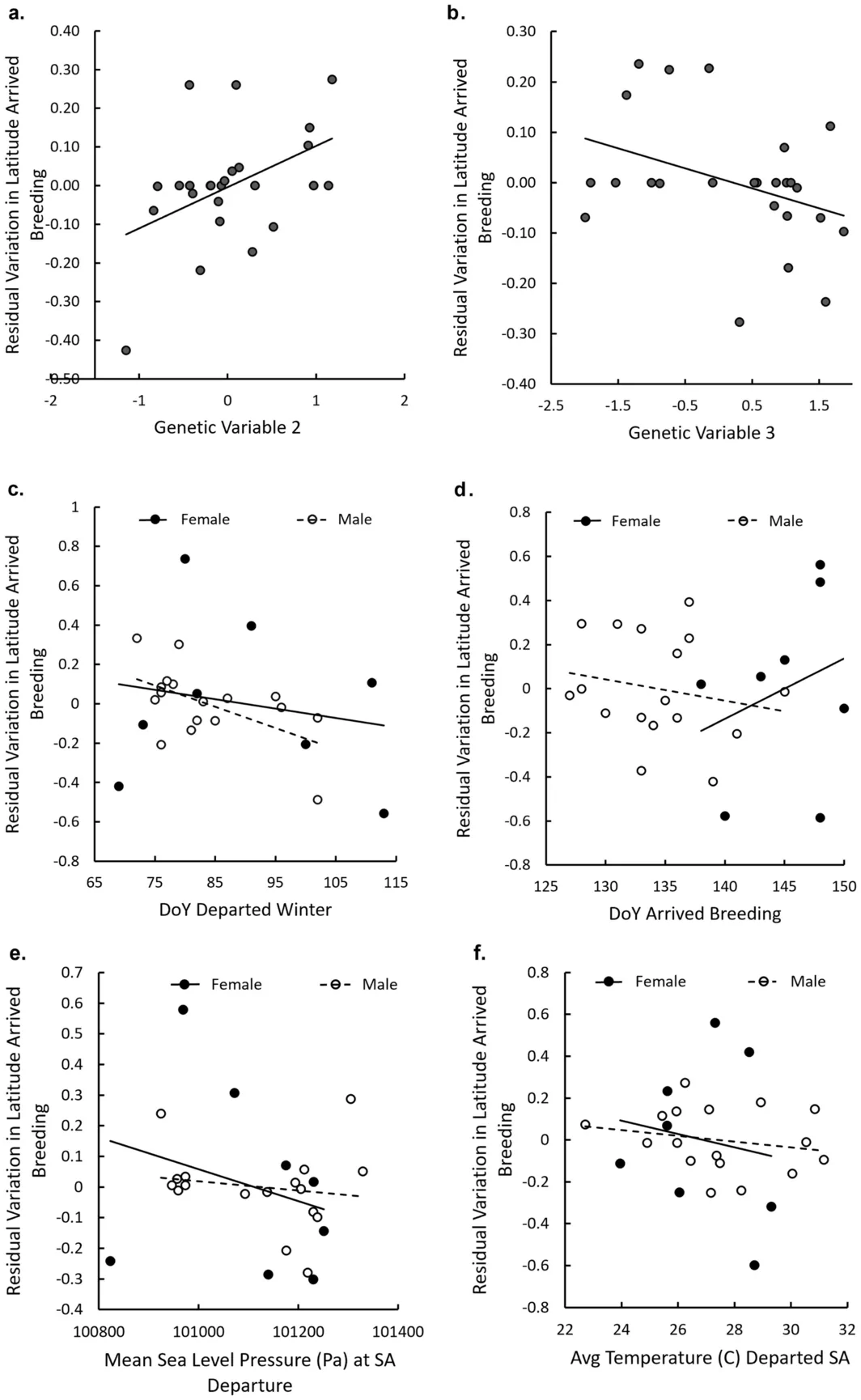
The location of Bobolink breeding ground arrival was correlated with both Genetic Variables 2 (a) and 3 (b), as well as the interaction between sex and DoY departed winter (c) and DoY arrived breeding ground (d). Weather had little effect on male location but females showed variation in MSL (e) and temperature (f). Residuals are the leftover variation in the response variable after the variation explained by all other explanatory variables besides the explanatory variable of interest has been factored out.

There were weak but significant genetic correlations with breeding ground latitude based on composite genotype scores 2 and 3, which collectively explained roughly 3% of latitudinal variation (Table S7, Figure 14a,b). While the majority of birds arrived at their breeding grounds near the latitude of our field site (44.5), 44% of females initially arrived at breeding grounds north of the geolocator error radius (46.0 N–46.5 N). Resultingly, the model identified a significant difference between latitudes of male and female arrival (Table S7). In the interaction between sex and start of spring migration, birds that went farther north were among the first to leave their wintering grounds, with the stronger trend seen in males (Figure 14c). In the interaction between sex and breeding ground DoY, the northern‐most females arrived among the latest to their breeding ground, whereas the opposite was true of males (Figure 14d). Interactions between sex and South American departure weather showed MSL (Figure 14e) and temperature (Figure 14f) had little effect on male breeding latitude but a stronger effect on female breeding latitude. Females driven by low pressure and temperature at their time of departure from South America ultimately settled at higher breeding latitudes, whereas little effect of weather was seen in males.

## 4 Discussion

Utilizing a combination of tracking, genetic, and climate reanalysis technologies, we found that genetics and sex correlated with migration events throughout the annual cycle in a long‐distance migrant songbird, the Bobolink. Our prediction that both the start and end migration dates and locations would be associated with genetic influences, was partially supported. Our genetic variables were correlated with the timing of four migration events including the start of fall migration and both the start and end of spring migration. Our genetic variables were only correlated withthe location of one event, the end of spring migration, and only weakly. Our prediction that during migration, timing and location would be largely influenced by weather conditions was partially supported. However, significant environmental effects from weather were not detected on any migration event unless they followed seasonal patterns (i.e., cooler weather at later dates in North American fall) or differences in timing or location between sexes. While some studies have compared the effects of specific genes on migration event behaviors (Bossu et al. 2022), to our knowledge, this is a rare study to document correlated genetic effects throughout the annual cycle using neutral genetic markers (Le Clercq et al. 2023).

### 4.1 Genetics

Our combined %PCA and MCOA approach provided three composite genetic variables with which to assess possible genetic influences on Bobolink migration dynamics, which collectively summarized 30% of the genetic variation in the population. These results are similar to other studies utilizing ordination analyses to summarize genetic structure in natural populations, which have reported 20%–45% of genetic variation summarized by the three most informative axes (e.g., Reeves and Richards 2009).

Our genetic variables were temporally correlated with four of seven major migration events, including the start and end dates of both fall and spring migration, as well as the date of arrival to South America. These results add to growing evidence that the timing of both spring and fall migration may be influenced by genetics (Bossu et al. 2022; Le Clercq et al. 2023), though most studies have historically focused on the timing of spring migration due to its direct effect on reproductive success in many species (Lemke et al. 2013; Helm and Liedvogel 2024). For Bobolinks, the role of genetics in the start of fall migration may help to assure that individuals are predisposed to leaving earlier for longer overwater flights. Genetics also helped to explain the arrival date to South America (Figure 1). The correlation between composite genotype scores and arrival date after an overwater flight further supports the hypothesis that endogenous controls are key in timing these risky migration events.

**FIGURE 15:**
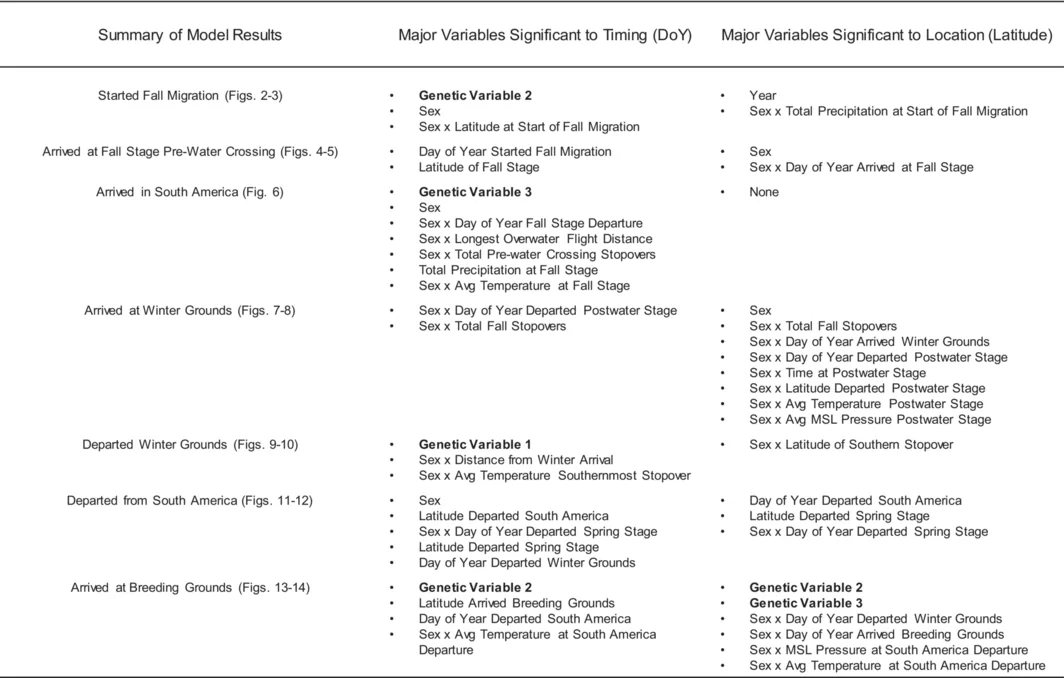
Summary of significant variables during each migration event in the study. Fall staging post‐water crossing and spring staging were not included as they were not directly studied as response variables in our study. Significant genetic variable results are highlighted in bold text.

Genetics also helped explain the latitude of breeding ground arrival, with this event detecting genetic correlations with two composite genotype scores within the same model, each likely representing correlated sets of loci. There was little variation in breeding ground location (43.65–46.8), with most birds returning to the breeding grounds where they were caught in Vermont (latitude 44.5). The higher number of migration events with genetically influenced timing versus location (Figure 15) indicates that heritable migration behavior in Bobolinks may be more closely linked to endogenous clock genes (Gwinner 1996; Butler 2003; Marra et al. 2005; Le Clercq et al. 2023) rather than migratory path route (Helbig 1991; Sokolovskis et al. 2023).

Arrival to fall pre‐water crossing stages, arrival at the wintering grounds at the end of fall migration, and departure from South America during spring migration were the only migration events in the annual cycle without a genetic correlation related to either timing or latitude. Before fall migration, 60% of birds exhibited a period of restlessness that went largely uncaptured in this study's design. This period of erratic movement may supplement staging and foraging behaviors before long over‐water flights, decreasing the influence of the stage we measured. Similarly, arrival to wintering grounds or departure from South America during spring migration may not necessitate endogenous systems if the bird is already properly fueled from staging or wintering. Future studies could further investigate this pre‐migration movement or stopover behavior to better understand the connection between genetics and fueling impacts on migration.

We could not directly measure the impacts of carryover effects on birth and/or survival rates. However, given that males departed South America before females and that later departure from the wintering grounds resulted in later arrival on the breeding grounds, there are likely direct effects on fitness (Marra et al. 1998). With Bobolink's polygynous mating system, early arrival on the breeding grounds is likely important for claiming high quality territories (Smith and Moore 2005) that can support > 1 female. Additionally, given the high rates of nest loss to hay harvest, a longer nesting season should provide more renesting opportunities. And, males that arrive on the breeding grounds after females will be at a disadvantage relative to territorial males in securing fertilizations (McKellar et al. 2013). The two other potential carryover effects were noted in females. Prior to fall migration, females staged farther north than males and arrived later than males on the wintering grounds. Additionally, the later females arrived in South America, the farther south they wintered, suggesting that there could be additional energetic costs for these females during spring migration. If endogenous cues activate to help birds mitigate risk during their migration, genetic signals may not be as necessary during these non‐breeding portions of the annual cycle. During spring migration, it is also possible that the reproductive cost of arriving late to the breeding grounds increases the importance of environmental cues to prepare for a water crossing.

### 4.2 Sex

There were consistent sex differences in both timing and location during several stages of the annual cycle. In many species, including Bobolinks (Renfrew et al. 2019), there is a reproductive advantage for protandry, when males arrive to breeding grounds earlier than females (Kokko et al. 2006; Lemke et al. 2013). Protandry tends to be stronger in populations with high rates of extra‐pair paternity (Kokko et al. 2006), which is common in our study population (White et al. 2022). We showed the continuation of the male‐first pattern throughout the entire annual cycle, indicating the influence of carry‐over effects between different portions of the cycle (Webster and Marra 2005). During fall migration, we observed later breeding ground departure in females, the majority of whom (78%) used breeding grounds as their pre‐water crossing fall staging grounds. Females may require more time to recover before migration due to their larger role in parental care (Webster and Marra 2005; Newton 2011; Lehikoinen and Santaharju 2017).

Sex played a role in latitudinal variation during fall migration, but was less determinant during spring migration. Spatial divergence between sexes, both during the pre‐water crossing fall stage and at wintering grounds, has not been previously observed in Bobolinks as it was assumed flocks were largely mixed sex (Renfrew et al. 2015). The most commonly used staging region in North America, 38 N, was a key location for males to congregate before flying approximately 1000 km over water to the Caribbean. Sexual segregation on nonbreeding grounds is common among migratory species (Bennett et al. 2019), but was previously undetected in Bobolinks because nonbreeding plumage is not sexually dimorphic (Renfrew et al. 2015). By the time birds departed wintering grounds to begin spring migration, there was no longer a significant difference between latitudes occupied by males and females.

At the breeding grounds, roughly half of all females used more northern breeding grounds on first arrival. While four out of the nine females in the study were recorded significantly overshooting the breeding grounds, the average difference between male and female breeding latitudes was within the error radius (1.2 ° latitude, 0.5 ° longitude) of our geolocators. More accurate tracking devices and larger sample sizes of tracked females are needed to further describe this behavior, particularly any associated benefits.

### 4.3 Environmental Effects

We modeled the temperature (°C), total precipitation (mm), and mean sea level pressure (Pa), that each bird experienced during its previous migration event. While these weather effects were significant in several of our migration event models (arrived in South America, departed wintering grounds, arrived at breeding grounds) the frequent interaction between sex showed that differences in timing or latitude between sexes often drove correlations. If weather determined a behavior related to timing or latitude, we would have detected either a consistent temperature within each migration event or warmer weather driving earlier migration behaviors consistent with broader migration phenology trends (Butler 2003; Marra et al. 2005; Saino et al. 2010). Instead, all temperature trends followed seasonal patterns for each region (e.g., warmer temperatures near the equator or cooler temperatures closer to winter). However, most birds avoided flying in the rain and experienced little (< 1 mm) to no rain before leaving a migration event. While there may have been significant differences in weather detected by our statistical models, the correlations most likely do not reflect migration decisions. Weather driven behavior may be undetectable at geolocator data spatial scale (error radius 1.2° latitude, 0.5° longitude) or occur outside of the migratory events investigated here.

Geolocator studies are inherently biased because they only include birds that successfully completed the full annual cycle. Much of the variation in migratory behavior and its influence on survival remains unknown because this technology cannot record failed or partial migrations in unsuccessful individuals. Experiments that directly controlled food resources for migrants showed that food availability may modulate the onset of endogenous cues to start spring migration (Stanley et al. 2022) and adjust the muscle/fat ratios necessary for timely spring migration (Cooper et al. 2015). Based on their successful migration, all birds in our study were sufficiently prepared to perform water crossings of the Gulf of Mexico, potentially downplaying the negative effects environmental conditions and food resources could have on Bobolink migration behavior.

This study is one of the first to test for the possible influences of genetic and environmental conditions on behaviors of a long‐distance migrant songbird across its whole annual cycle. Using microsatellite loci, light‐level geolocators, and climate reanalysis data, we detected correlations between genetics and major migration events. We found little evidence that Bobolinks utilized environmental conditions to determine their migratory behaviors. Instead, we found previously undocumented sexual differentiation in migration strategies between males and females during fall and spring migration. These spatial or temporal variations between males and females were responsible for the inconsistent impacts of environmental variables. As tracking technology continues to evolve, increased location accuracy and lighter device weights will improve our ability to determine location and weather conditions that birds experience throughout the annual cycle.

## Author Contributions

**Kathryn E. McGee:** conceptualization (equal), data curation (equal), formal analysis (lead), funding acquisition (supporting), investigation (equal), methodology (equal), validation (equal), visualization (equal), writing – original draft (equal), writing – review and editing (supporting). **Steven E. Travis:** formal analysis (equal), methodology (equal), supervision (equal), writing – original draft (equal), writing – review and editing (equal). **Allan M. Strong:** conceptualization (equal), project administration (equal), writing – review and editing (equal). **Noah G. Perlut:** conceptualization (equal), data curation (equal), formal analysis (equal), funding acquisition (lead), investigation (equal), methodology (equal), project administration (equal), resources (equal), supervision (equal), writing – original draft (equal), writing – review and editing (equal).

## Funding

This work was supported by D. Galipeau, U.S. Fish and Wildlife Service (F23AP01457‐00, F17AP006556), Nuttall Ornithological Club, Toyota TogetherGreen.

## Disclosure

Statement on Inclusion: Our study does not include scientists based in the country where the study animals migrated through.

## Conflicts of Interest

The authors declare no conflicts of interest.

## Supporting information


**Table S1:** Partial *R* ^2^ and ANOVA results for AIC selected models of start of fall migration.
**Table S2:** Partial *R* ^2^ and ANOVA results for AIC selected models of fall stage, pre‐water crossing.
**Table S3:** Partial *R* ^2^ and ANOVA results for AIC selected models of arrive to South America.
**Table S4:** Partial *R* ^2^ and ANOVA results for AIC selected models of arrive to winter grounds.
**Table S5:** Partial *R* ^2^ and ANOVA results for AIC selected models of depart wintering grounds.
**Table S6:** Partial *R* ^2^ and ANOVA results for AIC selected models of depart South America.
**Table S7:** Partial *R* ^2^ and ANOVA results for AIC selected models of breeding grounds arrival.
**Table S8:** Partial *R* ^2^ and ANOVA results for AIC selected models of depart wintering grounds.
**Table S9:** Partial *R* ^2^ and ANOVA results for AIC selected models of depart South America.
**Table S10:** Partial *R* ^2^ and ANOVA results for AIC selected models of breeding grounds arrival.
**Appendix S1:** Full multiple linear regression models run in R version 4.3.2.

**Data S1:** Processed geolocator dataset for all birds included in this study.

**Data S2:** Migration event dataset for all birds included in this study.

## Data Availability

All the required data are uploaded as Supporting Information.
