# Supplementary material for: Genetics, Not Weather, Informs Migration Behaviors in a Long‐Distance Migrant Songbird
Source: Ecol Evol. 2026 Sep 26;16(9):e74245. doi: 10.1002/ece3.74245 (PMC13615886; doi:10.1002/ece3.74245)
Supplement: Supplementary file 1 [file ECE3-16-e74245-s002.docx]

**Supplemental Tables:**

### Suppl. Table 1 Partial R^2^ and ANOVA results for AIC selected models of Start of Fall Migration

(Abbreviations: “x”= an interaction between the variables, DoY = Day of Year that the response variable being tested occurred, Temp=Temperature (C), TP= Total precipitation (mm), MSL= Mean sea level pressure (Pa), Gen=Genetic variable)

| **Response Variable:**  **DoY** | ANOVA  Df | ANOVA  F Value | ANOVA  P | Partial  R^2^ |
| --- | --- | --- | --- | --- |
| Gen2 | 1,19 | 9.329 | 0.007* | 0.224 |
| Sex | 1,19 | 10.338 | 0.005* | 0.060 |
| Latitude | 1,19 | 2.347 | 0.142 | 0.007 |
| MSL | 1,19 | 2.308 | 0.145 | 0.016 |
| Temp | 1,19 | 2.331 | 0.143 | 0.160 |
| TP | 1,19 | 0.142 | 0.710 | 0.002 |
| Sex x Latitude | 1,19 | 5.010 | 0.037* | 0.139 |
| Sex x MSL | 1,19 | 1.614 | 0.219 | 0.082 |
| Sex x TP | 1,19 | 3.560 | 0.075 | 0.035 |
| **R^2^** |  |  |  | **0.724** |
|  |  |  |  |  |
| **Response Variable:**  **Latitude** | ANOVA  Df | ANOVA  F Value | ANOVA  P | Partial  R^2^ |
| Gen2 | 1,11 | 1.849 | 0.201 | 0.014 |
| Gen3 | 1,11 | 1.848 | 0.201 | 0.028 |
| Year | 9,11 | 3.96 | 0.018* | 0.517 |
| Sex | 1,11 | 1.317 | 0.275 | 0.074 |
| DofY | 1,11 | 1.027 | 0.333 | 0.004 |
| TP | 1,11 | 0.031 | 0.864 | 0.024 |
| MSL | 1,11 | 1.384 | 0.264 | 0.01 |
| Sex x DoY | 1,11 | 1.88 | 0.198 | 0.08 |
| Sex x TP | 1,11 | 11.181 | 0.007* | 0.124 |
| **R^2^** |  |  |  | **0.876** |

### Suppl. Table 2 Partial R^2^ and ANOVA results for AIC selected models of Fall Stage, Pre-water crossing

(Abbreviations: “x”= an interaction between the variables, DoY = Day of Year that the response variable being tested occurred, Temp=Temperature (C), TP= Total precipitation (mm), MSL= Mean sea level pressure (Pa), Gen=Genetic variable)

| **Response Variable:**  **DoY** | ANOVA  Df | ANOVA  F Value | ANOVA  P | Partial  R^2^ |
| --- | --- | --- | --- | --- |
| Gen2 | 1,13 | 0.731 | 0.408 | 0.031 |
| Gen3 | 1,13 | 0.237 | 0.634 | 0.006 |
| Sex | 1,13 | 0.222 | 0.645 | 0.030 |
| Latitude | 1,13 | 20.691 | 0.001* | 0.119 |
| DoY_breeding_departure | 1,13 | 2.192 | 0.163 | 0.022 |
| DoY_start_fall | 1,13 | 5.019 | 0.043* | 0.119 |
| Temp_breeding_day | 1,13 | 1.039 | 0.327 | 0.008 |
| MSL_breeding_day | 1,13 | 0.018 | 0.894 | 0.012 |
| TP_breeding_day | 1,13 | 2.272 | 0.156 | 0.035 |
| Sex x Latitude | 1,13 | 0.432 | 0.523 | 0.128 |
| Sex x DoY_breeding_departure | 1,13 | 0.066 | 0.802 | 0.033 |
| Sex x DoY_start_fall | 1,13 | 2.063 | 0.175 | 0.159 |
| Sex x Temp_breeding_day | 1,13 | 2.686 | 0.125 | 0.037 |
| Sex x MSL_breeding_day | 1,13 | 0.520 | 0.483 | 0.041 |
| Sex x TP_breeding_day | 1,13 | 1.058 | 0.322 | 0.062 |
| **R^2^** |  |  |  | **0.842** |
|  |  |  |  |  |
| **Response Variable:**  **Latitude** | ANOVA  Df | ANOVA  F Value | ANOVA  P | Partial  R^2^ |
| Gen1 | 1,12 | 0.097 | 0.761 | 0.010 |
| Gen2 | 1,12 | 0.710 | 0.416 | 0.013 |
| Gen3 | 1,12 | 0.582 | 0.460 | 0.006 |
| Sex | 1,12 | 21.576 | 0.001* | 0.055 |
| Sex x DoY | 1,12 | 16.018 | 0.000* | 0.417 |
| Sex x DoY_breeding_departure | 2,12 | 1.196 | 0.336 | 0.084 |
| Sex x DoY_start_fall | 2,12 | 1.865 | 0.197 | 0.091 |
| Sex x Temp_breeding_day | 2,12 | 0.248 | 0.784 | 0.071 |
| Sex x MSL_breeding_day | 2,12 | 0.026 | 0.975 | 0.063 |
| Sex x TP_breeding_day | 2,12 | 0.095 | 0.910 | 0.021 |
| **R^2^** |  |  |  | **0.833** |

### Suppl. Table 3 Partial R^2^ and ANOVA results for AIC selected models of Arrive to South America

(Abbreviations: “x”= an interaction between the variables, Avg= Average, DoY = Day of Year that the response variable being tested occurred, Temp= Temperature (C), TP= Total precipitation (mm), MSL= Mean sea level pressure (Pa), Gen=Genetic variable)

| **Response Variable:**  **DoY** | ANOVA  Df | ANOVA  F Value | ANOVA  P | Partial R^2^ |
| --- | --- | --- | --- | --- |
| Gen3 | 1,15 | 13.171 | 0.002* | 0.053 |
| Sex | 1,15 | 15.064 | 0.001* | 0.110 |
| Avg_Temp_Stage | 1,15 | 1.320 | 0.269 | 0.012 |
| Avg_TP_Stage | 1,15 | 5.409 | 0.034* | 0.116 |
| Longest Overwater Flight Distance | 1,15 | 4.908 | 0.043* | 0.019 |
| Total Stopover Pre-water Flight | 1,15 | 0.289 | 0.599 | 0.003 |
| DoY Start Fall | 1,15 | 1.334 | 0.266 | 0.025 |
| DoY Pre-water Stage Departure | 1,15 | 18.439 | 0.001* | 0.055 |
| Sex x Avg_Temp_Stage | 1,15 | 5.556 | 0.032* | 0.054 |
| Sex x Longest_Overwater_Flight_Distance | 1,15 | 33.701 | 0.000* | 0.155 |
| Sex x Total_Stopover_Pre-water | 1,15 | 16.355 | 0.001* | 0.094 |
| Sex x DoY_Start_Fall | 1,15 | 2.485 | 0.136 | 0.082 |
| Sex x DoY_Pre-water_Stage_Departure | 1,15 | 14.407 | 0.002* | 0.131 |
| **R^2^** |  |  |  | **0.908** |
|  |  |  |  |  |
| **Response Variable:**  **Latitude** | ANOVA  Df | ANOVA  F Value | ANOVA  P | Partial R^2^ |
| Gen2 | 1,10 | 1.195 | 0.300 | 0.018 |
| Gen3 | 1,10 | 2.117 | 0.176 | 0.025 |
| Sex | 2,10 | 0.016 | 0.984 | 0.032 |
| DoY | 1,10 | 0.119 | 0.737 | 0.006 |
| Total_Stopover_NA | 1,10 | 0.682 | 0.428 | 0.026 |
| DoY_Pre-water_Stage_Departure | 1,10 | 1.395 | 0.265 | 0.040 |
| DoY_Start_Fall | 1,10 | 0.405 | 0.539 | 0.022 |
| Longest_Overwater_Flight_Distance | 1,10 | 0.513 | 0.490 | 0.014 |
| Avg_Temp_Stage | 1,10 | 0.182 | 0.679 | 0.011 |
| Avg_MSL_Stage | 1,10 | 1.497 | 0.249 | 0.024 |
| Avg_TP_Stage | 1,10 | 0.002 | 0.964 | 0.016 |
| Sex x DoY | 1,10 | 2.368 | 0.155 | 0.045 |
| Sex x Total_Stopover_NA | 1,10 | 3.705 | 0.083 | 0.056 |
| Sex x DoY_Pre-water_Stage_Departure | 1,10 | 2.983 | 0.115 | 0.068 |
| Sex x DoY_Start_Fall | 1,10 | 3.082 | 0.110 | 0.059 |
| Sex x Longest_Overwater_Flight_Distance | 1,10 | 2.615 | 0.137 | 0.034 |
| Sex x Avg_Temp_Stage | 1,10 | 3.139 | 0.107 | 0.054 |
| Sex x Avg_TP_Stage | 1,10 | 1.214 | 0.296 | 0.105 |
| **R^2^** |  |  |  | **0.656** |

### Suppl. Table 4 Partial R^2^ and ANOVA results for AIC selected models of Arrive to Winter Grounds

(Model abbreviations: “x”= an interaction between the variables, Avg=Average, DoY = Day of Year that the response variable being tested occurred, Lat= Latitude Temp= Temperature (C), TP= Total precipitation (mm), MSL= Mean sea level pressure (Pa), Gen=Genetic variable)

| **Response Variable:**  **DoY** | ANOVA  Df | ANOVA  F Value | ANOVA  P | Partial R^2^ |
| --- | --- | --- | --- | --- |
| Gen1 | 1,15 | 1.846 | 0.194 | 0.046 |
| Sex | 1,15 | 3.321 | 0.088 | 0.087 |
| Avg_MSL_Postwater_Stage | 1,15 | 0.814 | 0.381 | 0.013 |
| Avg_Temp_Postwater_Stage | 1,15 | 0.596 | 0.452 | 0.011 |
| Total_Fall_Stopovers | 1,15 | 1.248 | 0.281 | 0.006 |
| DoY_Depart_Postwater_Stage | 1,15 | 4.249 | 0.057 | 0.105 |
| Time_Postwater_Stage | 1,15 | 4.538 | 0.050* | 0.025 |
| Lat_Depart_Postwater_Stage | 1,15 | 0.077 | 0.785 | 0.011 |
| Sex x Avg_MSL_Postwater_Stage | 1,15 | 2.901 | 0.109 | 0.094 |
| Sex x Avg_Temp_Postwater_Stage | 1,15 | 1.123 | 0.306 | 0.07 |
| Sex x Total_Fall_Stopovers | 1,15 | 5.080 | 0.040* | 0.085 |
| Sex x DoY_Depart_Postwater_Stage | 1,15 | 5.426 | 0.034* | 0.209 |
| Sex x Lat_Depart_Postwater_Stage | 1,15 | 1.421 | 0.252 | 0.053 |
| **R^2^** |  |  |  | **0.815** |
|  |  |  |  |  |
| **Response Variable:**  **Latitude** | ANOVA  Df | ANOVA  F Value | ANOVA  P | Partial R^2^ |
| Gen1 | 1,11 | 0.799 | 0.391 | 0.009 |
| Gen3 | 1,11 | 1.007 | 0.337 | 0.011 |
| Sex | 1,11 | 9.929 | 0.009* | 0.077 |
| DoY | 1,11 | 0.007 | 0.934 | 0.014 |
| DoY_Depart_Postwater_Stage | 1,11 | 2.860 | 0.119 | 0.018 |
| Total_Fall_Stopovers | 1,11 | 3.357 | 0.094 | 0.014 |
| Time_Postwater_Stage | 1,11 | 1.782 | 0.209 | 0.007 |
| Lat_Depart_Postwater_Stage | 1,11 | 0.284 | 0.605 | 0.015 |
| Avg_Temp_Postwater_Stage | 1,11 | 2.028 | 0.182 | 0.032 |
| Avg_MSL_Postwater_Stage | 1,11 | 1.294 | 0.279 | 0.012 |
| Sex x DoY | 1,11 | 18.280 | 0.001* | 0.097 |
| Sex x DoY_Depart_Postwater_Stage | 1,11 | 19.058 | 0.001* | 0.100 |
| Sex x Total_Fall_Stopovers | 1,11 | 19.174 | 0.001* | 0.095 |
| Sex x Time_Postwater_Stage | 1,11 | 14.506 | 0.003* | 0.06 |
| Sex x Lat_Depart_Postwater_Stage | 1,11 | 11.339 | 0.006* | 0.062 |
| Sex x Avg_Temp_Postwater_Stage | 1,11 | 6.873 | 0.024* | 0.088 |
| Sex x Avg_MSL_Postwater_Stage | 1,11 | 19.104 | 0.001* | 0.092 |
| **R^2^** |  |  |  | **0.803** |

### Suppl. Table 5 Partial R^2^and ANOVA results for AIC selected models of Depart Wintering Grounds

(Model abbreviations: “x”= an interaction between the variables, Avg=Average, Lat= Latitude, DoY = Day of Year that the response variable being tested occurred, Southstop= Southern-most stopover location in wintering grounds, Temp=Temperature (C), TP= Total precipitation (mm), MSL= Mean sea level pressure (Pa), Gen=Genetic variable)

| **Response Variable:**  **DoY** | ANOVA  Df | ANOVA  F Value | ANOVA  P | Partial  R^2^ |
| --- | --- | --- | --- | --- |
| Gen1 | 1,11 | 22.686 | 0.001* | 0.105 |
| Gen2 | 1,11 | 4.461 | 0.058 | 0.04 |
| Sex | 1,11 | 0.003 | 0.959 | 0.041 |
| DoY_Southern_Stopover | 1,11 | 49.930 | 0.000* | 0.408 |
| Lat_Southern_Stopover | 1,11 | 1.036 | 0.331 | 0.015 |
| Latitude | 1,11 | 2.016 | 0.183 | 0.015 |
| Time_Southern_Stopover_hours | 1,11 | 0.000 | 0.984 | 0.005 |
| Distance_from_Winter_Arrival | 1,11 | 4.008 | 0.071 | 0.035 |
| Avg_Temp_Southern_Stopover | 1,11 | 7.981 | 0.017* | 0.019 |
| Sex x Lat_Southern_Stopover | 1,11 | 3.137 | 0.104 | 0.035 |
| Sex x Latitude | 1,11 | 3.741 | 0.079 | 0.037 |
| Sex x Time_Southern_Stopover_hours | 1,11 | 2.115 | 0.174 | 0.02 |
| Sex x Distance_from_Winter_Arrival | 1,11 | 5.760 | 0.035* | 0.059 |
| Sex x Avg_Temp_Southern_Stopover | 1,11 | 7.379 | 0.020* | 0.055 |
| **R^2^** |  |  |  | **0.887** |
|  |  |  |  |  |
| **Response Variable:**  **Latitude** | ANOVA  Df | ANOVA  F Value | ANOVA  P | Partial R^2^ |
| Gen1 | 1,8 | 0.955 | 0.357 | 0.001 |
| Year | 8,8 | 2.215 | 0.141 | 0.114 |
| Sex | 1,8 | 0.01 | 0.922 | 0.01 |
| Avg_Temp_Southern_Stopover | 1,8 | 1.262 | 0.294 | 0.087 |
| DoY_Southern_Stopover | 1,8 | 3.114 | 0.116 | 0.011 |
| Lat_Southern_Stopover | 1,8 | 414.915 | 0.000* | 0.236 |
| Time_Southern_Stopover_hours | 1,8 | 1.632 | 0.237 | 0.029 |
| Distance_from_Winter_Arrival | 1,8 | 2.224 | 0.174 | 0.133 |
| Sex x Lat_Southern_Stopover | 1,8 | 9.724 | 0.014* | 0.237 |
| Sex x Distance_from_Winter_Arrival | 1,8 | 4.574 | 0.065 | 0.14 |
| **R^2^** |  |  |  | **0.998** |

### Suppl. Table 6 Partial R^2^ and ANOVA results for AIC selected models of Depart South America

(Model abbreviations: “x”= an interaction between the variables, Lat= Latitude, DoY = Day of Year that the response variable being tested occurred, Temp =Temperature (C), TP= Total precipitation (mm), MSL= Mean sea level pressure (Pa), Gen=Genetic variable)

| **Response Variable:**  **DoY** | ANOVA  Df | ANOVA  F Value | ANOVA  P | Partial  R^2^ |
| --- | --- | --- | --- | --- |
| Gen2 | 1,15 | 1.610 | 0.224 | 0.004 |
| Sex | 1,15 | 27.696 | 0.000* | 0.179 |
| Latitude | 1,15 | 13.984 | 0.002* | 0.044 |
| DoY_Spring_Stage_Departure | 1,15 | 52.642 | 0.000* | 0.192 |
| Lat_Spring_Stage_Departure | 1,15 | 30.041 | 0.000* | 0.038 |
| DoY_Depart_Winter | 1,15 | 5.094 | 0.039* | 0.019 |
| Sex x DoY_Spring_Stage_Departure | 1,15 | 7.615 | 0.015* | 0.248 |
| Sex x Lat_Spring_Stage_Departure | 1,15 | 2.160 | 0.162 | 0.046 |
| Sex x DoY_Depart_Winter | 1,15 | 3.940 | 0.066 | 0.167 |
| **R^2^** |  |  |  | **0.937** |
|  |  |  |  |  |
| **Response Variable:**  **Latitude** | ANOVA  Df | ANOVA  F Value | ANOVA  P | Partial R^2^ |
| Sex | 1,17 | 5.039 | 0.038* | 0.045 |
| DoY | 1,17 | 13.614 | 0.002* | 0.188 |
| DoY_Spring_Stage_Departure | 1,17 | 7.112 | 0.016* | 0.018 |
| DoY_Depart_Winter | 1,17 | 2.843 | 0.110 | 0.007 |
| Lat_Spring_Stage_Departure | 1,17 | 9.457 | 0.007* | 0.115 |
| Sex x DoY_Spring_Stage_Departure | 1,17 | 5.351 | 0.033* | 0.082 |
| Sex x DoY_Depart_Winter | 1,17 | 2.652 | 0.122 | 0.055 |
| **R^2^** |  |  |  | **0.511** |

### Suppl. Table 7 Partial R^2^ and ANOVA results for AIC selected models of Breeding Grounds Arrival

(Model abbreviations: “x”= an interaction between the variables, NA= North America, SA=South America, Lat= Latitude, DoY = Day of Year that the response variable being tested occurred, Temp= Temperature (C), TP= Total precipitation (mm), MSL= Mean sea level pressure (Pa), Gen=Genetic variable)

| **Response Variable:**  **DoY** | ANOVA  Df | ANOVA  F Value | ANOVA  P | Partial  R^2^ |
| --- | --- | --- | --- | --- |
| Gen2 | 1,13 | 5.252 | 0.039* | 0.016 |
| Sex | 1,13 | 1.300 | 0.275 | 0.092 |
| DoY_SA_Departure | 1,13 | 46.202 | 0.000* | 0.322 |
| DoY_Winter_Departure | 1,13 | 5.059 | 0.042* | 0.020 |
| Latitude | 1,13 | 5.602 | 0.034* | 0.052 |
| MSL_SA_Departure | 1,13 | 0.154 | 0.701 | 0.012 |
| Temp_SA_Departure | 1,13 | 0.016 | 0.903 | 0.009 |
| Sex x DoY_Winter_Departure | 1,13 | 2.599 | 0.131 | 0.096 |
| Sex x Latitude | 1,13 | 1.876 | 0.194 | 0.107 |
| Sex x MSL_SA_Departure | 1,13 | 1.188 | 0.295 | 0.101 |
| Sex x Temp_SA_Departure | 1,13 | 8.091 | 0.014* | 0.121 |
| **R^2^** |  |  |  | **0.946** |
|  |  |  |  |  |
| **Response Variable:**  **Latitude** | ANOVA  Df | ANOVA  F Value | ANOVA  P | Partial  R^2^ |
| Gen1 | 1,6 | 1.181 | 0.319 | 0.024 |
| Gen2 | 1,6 | 11.372 | 0.015* | 0.016 |
| Gen3 | 1,6 | 6.742 | 0.041* | 0.014 |
| Sex | 1,6 | 21.596 | 0.004* | 0.070 |
| Total_Stopover_NA_Spring | 1,6 | 9.847 | 0.020* | 0.007 |
| DoY | 1,6 | 3.603 | 0.106 | 0.049 |
| DoY_SA_Departure | 1,6 | 6.369 | 0.045* | 0.030 |
| DoY_Winter_Departure | 1,6 | 28.848 | 0.002* | 0.053 |
| MSL_SA_Departure | 1,6 | 9.384 | 0.022* | 0.014 |
| Temp_SA_Departure | 1,6 | 11.428 | 0.015* | 0.006 |
| TP_SA_Departure | 1,6 | 2.002 | 0.207 | 0.003 |
| Sex x Total_stop_NA_spring | 1,6 | 0.987 | 0.359 | 0.124 |
| Sex x DoY | 1,6 | 64.671 | 0.000* | 0.153 |
| Sex x DoY_SA_Departure | 1,6 | 2.243 | 0.185 | 0.077 |
| Sex x DoY_Winter_Departure | 1,6 | 24.113 | 0.003* | 0.137 |
| Sex x MSL_SA_Departure | 1,6 | 16.237 | 0.007* | 0.085 |
| Sex x Temp_SA_Departure | 1,6 | 29.945 | 0.002* | 0.085 |
| Sex x TP_SA_Departure | 1,6 | 0.922 | 0.374 | 0.039 |
| **R^2^** |  |  |  | **0.986** |

### Suppl. Table 8 Partial R^2^and ANOVA results for AIC selected models of Depart Wintering Grounds

(Model abbreviations: “x”= an interaction between the variables, Avg=Average, Lat= Latitude, DoY = Day of Year that the response variable being tested occurred, Southstop= Southern-most stopover location in wintering grounds, Temp=Temperature (C), TP= Total precipitation (mm), MSL= Mean sea level pressure (Pa), Gen=Genetic variable)

| **Response Variable:**  **DoY** | ANOVA  Df | ANOVA  F Value | ANOVA  P | Partial  R^2^ |
| --- | --- | --- | --- | --- |
| Gen1 | 1,11 | 22.686 | 0.001* | 0.105 |
| Gen2 | 1,11 | 4.461 | 0.058 | 0.04 |
| Sex | 1,11 | 0.003 | 0.959 | 0.041 |
| DoY_Southern_Stopover | 1,11 | 49.930 | 0.000* | 0.408 |
| Lat_Southern_Stopover | 1,11 | 1.036 | 0.331 | 0.015 |
| Latitude | 1,11 | 2.016 | 0.183 | 0.015 |
| Time_Southern_Stopover_hours | 1,11 | 0.000 | 0.984 | 0.005 |
| Distance_from_Winter_Arrival | 1,11 | 4.008 | 0.071 | 0.035 |
| Avg_Temp_Southern_Stopover | 1,11 | 7.981 | 0.017* | 0.019 |
| Sex x Lat_Southern_Stopover | 1,11 | 3.137 | 0.104 | 0.035 |
| Sex x Latitude | 1,11 | 3.741 | 0.079 | 0.037 |
| Sex x Time_Southern_Stopover_hours | 1,11 | 2.115 | 0.174 | 0.02 |
| Sex x Distance_from_Winter_Arrival | 1,11 | 5.760 | 0.035* | 0.059 |
| Sex x Avg_Temp_Southern_Stopover | 1,11 | 7.379 | 0.020* | 0.055 |
| **R^2^** |  |  |  | **0.887** |
|  |  |  |  |  |
| **Response Variable:**  **Latitude** | ANOVA  Df | ANOVA  F Value | ANOVA  P | Partial R^2^ |
| Gen1 | 1,8 | 0.955 | 0.357 | 0.001 |
| Year | 8,8 | 2.215 | 0.141 | 0.114 |
| Sex | 1,8 | 0.01 | 0.922 | 0.01 |
| Avg_Temp_Southern_Stopover | 1,8 | 1.262 | 0.294 | 0.087 |
| DoY_Southern_Stopover | 1,8 | 3.114 | 0.116 | 0.011 |
| Lat_Southern_Stopover | 1,8 | 414.915 | 0.000* | 0.236 |
| Time_Southern_Stopover_hours | 1,8 | 1.632 | 0.237 | 0.029 |
| Distance_from_Winter_Arrival | 1,8 | 2.224 | 0.174 | 0.133 |
| Sex x Lat_Southern_Stopover | 1,8 | 9.724 | 0.014* | 0.237 |
| Sex x Distance_from_Winter_Arrival | 1,8 | 4.574 | 0.065 | 0.14 |
| **R^2^** |  |  |  | **0.998** |

### Suppl. Table 9 Partial R^2^ and ANOVA results for AIC selected models of Depart South America

(Model abbreviations: “x”= an interaction between the variables, Lat= Latitude, DoY = Day of Year that the response variable being tested occurred, Temp =Temperature (C), TP= Total precipitation (mm), MSL= Mean sea level pressure (Pa), Gen=Genetic variable)

| **Response Variable:**  **DoY** | ANOVA  Df | ANOVA  F Value | ANOVA  P | Partial  R^2^ |
| --- | --- | --- | --- | --- |
| Gen2 | 1,15 | 1.610 | 0.224 | 0.004 |
| Sex | 1,15 | 27.696 | 0.000* | 0.179 |
| Latitude | 1,15 | 13.984 | 0.002* | 0.044 |
| DoY_Spring_Stage_Departure | 1,15 | 52.642 | 0.000* | 0.192 |
| Lat_Spring_Stage_Departure | 1,15 | 30.041 | 0.000* | 0.038 |
| DoY_Depart_Winter | 1,15 | 5.094 | 0.039* | 0.019 |
| Sex x DoY_Spring_Stage_Departure | 1,15 | 7.615 | 0.015* | 0.248 |
| Sex x Lat_Spring_Stage_Departure | 1,15 | 2.160 | 0.162 | 0.046 |
| Sex x DoY_Depart_Winter | 1,15 | 3.940 | 0.066 | 0.167 |
| **R^2^** |  |  |  | **0.937** |
|  |  |  |  |  |
| **Response Variable:**  **Latitude** | ANOVA  Df | ANOVA  F Value | ANOVA  P | Partial R^2^ |
| Sex | 1,17 | 5.039 | 0.038* | 0.045 |
| DoY | 1,17 | 13.614 | 0.002* | 0.188 |
| DoY_Spring_Stage_Departure | 1,17 | 7.112 | 0.016* | 0.018 |
| DoY_Depart_Winter | 1,17 | 2.843 | 0.110 | 0.007 |
| Lat_Spring_Stage_Departure | 1,17 | 9.457 | 0.007* | 0.115 |
| Sex x DoY_Spring_Stage_Departure | 1,17 | 5.351 | 0.033* | 0.082 |
| Sex x DoY_Depart_Winter | 1,17 | 2.652 | 0.122 | 0.055 |
| **R^2^** |  |  |  | **0.511** |

### Suppl. Table 10 Partial R^2^ and ANOVA results for AIC selected models of Breeding Grounds Arrival

(Model abbreviations: “x”= an interaction between the variables, NA= North America, SA=South America, Lat= Latitude, DoY = Day of Year that the response variable being tested occurred, Temp= Temperature (C), TP= Total precipitation (mm), MSL= Mean sea level pressure (Pa), Gen=Genetic variable)

| **Response Variable:**  **DoY** | ANOVA  Df | ANOVA  F Value | ANOVA  P | Partial  R^2^ |
| --- | --- | --- | --- | --- |
| Gen2 | 1,13 | 5.252 | 0.039* | 0.016 |
| Sex | 1,13 | 1.300 | 0.275 | 0.092 |
| DoY_SA_Departure | 1,13 | 46.202 | 0.000* | 0.322 |
| DoY_Winter_Departure | 1,13 | 5.059 | 0.042* | 0.020 |
| Latitude | 1,13 | 5.602 | 0.034* | 0.052 |
| MSL_SA_Departure | 1,13 | 0.154 | 0.701 | 0.012 |
| Temp_SA_Departure | 1,13 | 0.016 | 0.903 | 0.009 |
| Sex x DoY_Winter_Departure | 1,13 | 2.599 | 0.131 | 0.096 |
| Sex x Latitude | 1,13 | 1.876 | 0.194 | 0.107 |
| Sex x MSL_SA_Departure | 1,13 | 1.188 | 0.295 | 0.101 |
| Sex x Temp_SA_Departure | 1,13 | 8.091 | 0.014* | 0.121 |
| **R^2^** |  |  |  | **0.946** |
|  |  |  |  |  |
| **Response Variable:**  **Latitude** | ANOVA  Df | ANOVA  F Value | ANOVA  P | Partial  R^2^ |
| Gen1 | 1,6 | 1.181 | 0.319 | 0.024 |
| Gen2 | 1,6 | 11.372 | 0.015* | 0.016 |
| Gen3 | 1,6 | 6.742 | 0.041* | 0.014 |
| Sex | 1,6 | 21.596 | 0.004* | 0.070 |
| Total_Stopover_NA_Spring | 1,6 | 9.847 | 0.020* | 0.007 |
| DoY | 1,6 | 3.603 | 0.106 | 0.049 |
| DoY_SA_Departure | 1,6 | 6.369 | 0.045* | 0.030 |
| DoY_Winter_Departure | 1,6 | 28.848 | 0.002* | 0.053 |
| MSL_SA_Departure | 1,6 | 9.384 | 0.022* | 0.014 |
| Temp_SA_Departure | 1,6 | 11.428 | 0.015* | 0.006 |
| TP_SA_Departure | 1,6 | 2.002 | 0.207 | 0.003 |
| Sex x Total_stop_NA_spring | 1,6 | 0.987 | 0.359 | 0.124 |
| Sex x DoY | 1,6 | 64.671 | 0.000* | 0.153 |
| Sex x DoY_SA_Departure | 1,6 | 2.243 | 0.185 | 0.077 |
| Sex x DoY_Winter_Departure | 1,6 | 24.113 | 0.003* | 0.137 |
| Sex x MSL_SA_Departure | 1,6 | 16.237 | 0.007* | 0.085 |
| Sex x Temp_SA_Departure | 1,6 | 29.945 | 0.002* | 0.085 |
| Sex x TP_SA_Departure | 1,6 | 0.922 | 0.374 | 0.039 |
| **R^2^** |  |  |  | **0.986** |

## Appendix

### Appendix 1. Full multiple linear regression models run in R version 4.3.2.

(Model abbreviations: lm=linear model, “x”= an interaction between the variables, Avg=Average, NA= North America, SA=South America, LAT= Latitude, DoY = Day of Year that the response variable being tested occurred, Southstop= Southern-most stopover location in wintering grounds, Temp=Temperature (C), TP= Total precipitation (mm), MSL= Mean sea level pressure (Pa), Gen=Genetic variable)

| **Migration Event** | **Full Model** |
| --- | --- |
| **Started Fall Migration:**  ***Day of Year*** | START_FALL_DoY <- lm(DoY ~ Gen1 + Gen2 + Gen3 + Sex + Latitude + MSL + Temp + TP+ Sex x Latitude + Sex x MSL + Sex x Temp+ Sex x TP) |
| **Started Fall Migration:**  ***Latitude*** | START_FALL_LAT <- lm(Latitude ~ Gen1 + Gen2 + Gen3 + Year + Sex + DoY + TP + Temp + MSL + Sex x DoY + Sex x TP + Sex x Temp+ Sex x MSL) |
| **Arrived at Fall Stage, Pre-Water Crossing:**  ***Day of Year*** | FALL_STAGING_DoY <-lm(DoY ~ Gen1 + Gen2 + Gen3 + Sex +Latitude + Distance_from_Breeding_Ground_lat + DoY_Breeding_Ground_Departure + DoY_Start_Fall + Temp_Breeding_Ground_Departure + MSL_Breeding_Ground_Departure + TP_Breeding_Ground_Departure + Sex x Latitude + Sex x Distance_from_Breeding_Ground_lat + Sex x DoY_Breeding_Ground_Departure + Sex x DoY_Start_Fall + Sex x Temp_Breeding_Ground_Departure + Sex x MSL_Breeding_Ground_Departure + Sex x TP_Breeding_Ground_Departure) |
| **Arrived at Fall Stage Pre-Water Crossing:**  ***Latitude*** | FALL_STAGING_LAT <-lm(Latitude ~ Gen1 + Gen2 + Gen3 + Sex +DoY +DoY_Breeding_Ground_Departure + DoY_Start_Fall + Temp_Breeding_Ground + MSL_Breeding_Ground + TP_Breeding_Ground + Sex x DoY + Sex x DoY_Breeding_Ground_Departure + Sex x DoY_Start_Fall + Sex x Temp_Breeding_Ground + Sex x MSL_Breeding_Ground + Sex x TP_Breeding_Ground) |
| **Arrived in South America:**  ***Day of Year*** | ARRIVE_SA_DoY <-lm(DoY ~ Gen1 + Gen2 + Gen3 + Sex +Avg_Temp_Stage + Avg_MSL_Stage + Avg_TP_Stage + Latitude + Longest_Overwater_Flight_Distance + DoY_Start_Fall + DoY_Pre-water_Stage_Departure + Total_Stopover_NA + Sex x Avg_Temp_Stage + Sex x Avg_MSL_Stage + Sex x Avg_TP_Stage + Sex x Latitude + Sex x Longest_Overwater_Flight_Distance + Sex x DoY_Start_Fall + Sex x DoY_Pre-water_Stage_Departure + Sex x Total_Stopover_NA) |
| **Arrived in South America:**  ***Latitude*** | ARRIVE_SA_LAT <-lm(Latitude ~ Gen1 + Gen2 + Gen3 + Sex +DoY + Total_Stopover_NA + DoY_Pre-water_Stage_Departure + DoY_Start_Fall + Longest_Overwater_Flight_Distance + Avg_Temp_Stage + Avg_MSL_Stage + Avg_TP_Stage+ Sex x DoY + Sex x Total_Stopover_NA + Sex x DoY_Pre-water_Stage_Departure + Sex x DoY_Start_Fall + Sex x Longest_Overwater_Flight_Distance + Sex x Avg_Temp_Stage + Sex x Avg_MSL_Stage + Sex x Avg_TP_Stage) |
| **Arrived Winter Grounds:**  ***Day of Year*** | ARRIVE_WINTER_DoY <-lm(DoY ~ Gen1 + Gen2 + Gen3 + Sex + Avg_MSL_Postwater_Stage + Avg_Temp_Postwater_Stage + Avg_TP_Postwater_Stage + Total_Fall_Stopover +  DoY_Depart_Postwater_Stage + Time_Postwater_Stage + Lat_Depart_Postwater_Stage + Latitude Sex x Avg_MSL_Postwater_Stage + Sex x Avg_Temp_Postwater_Stage + Sex x Avg_TP_Postwater_Stage + Sex x Total_Fall_Stopover +  Sex x DoY_Depart_Postwater_Stage + Sex x Time_Postwater_Stage + Sex x Lat_Depart_Postwater_Stage + Sex x Latitude) |
| **Arrived Winter Grounds:**  ***Latitude*** | ARRIVE_WINTER_LAT <-lm(Latitude ~ Gen1 + Gen2 + Gen3 + Sex + Total_Fall_Stopover + DoY + DoY_Depart_Postwater_Stage + Time_Postwater_Stage + Lat_Depart_Postwater_Stage + Avg_Temp_Postwater_Stage + Avg_MSL_Postwater_Stage + Avg_TP_Postwater_Stage +Sex x Total_Fall_Stopover + Sex x DoY + Sex x DoY_Depart_Postwater_Stage + Sex x Time_Postwater_Stage + Sex x Lat_Depart_Postwater_Stage + Sex x Avg_Temp_Postwater_Stage + Sex x Avg_MSL_Postwater_Stage + Sex x Avg_TP_Postwater_Stage) |
| **Departed Winter Grounds:**  ***Day of Year*** | DEPART_WINTER_DoY <-lm(DoY ~ Gen1 + Gen2 + Gen3 + Sex +DoY_Southstop + Lat_Southstop + Latitude + Time_Southstop_hours + Distance_from_Winter_Arrival + Avg_MSL_Southstop + Avg_Temp_Southstop + Avg_TP_Southstop +Sex x DoY_Southstop + Sex x Lat_Southstop + Sex x Latitude + Sex x Time_Southstop_hours + Sex x Distance_from_Winter_Arrival + Sex x Avg_MSL_Southstop + Sex x Avg_Temp_Southstop + Sex x Avg_TP_Southstop) |
| **Departed Winter Grounds:**  ***Latitude*** | DEPART_WINTER_LAT <-lm(Latitude ~ Gen1 + Gen2 + Gen3 + Year + Avg_Temp_Southstop + Avg_MSL_Southstop + Avg_TP_Southstop + Sex +DoY_Southstop + Lat_Southstop + Southstop_Total_Hours + Distance_from_Winter_Arrival +Sex x DoY_Southstop + Sex x Lat_Southstop + Sex x Southstop_Total_Hours + Sex x Distance_from_Winter_Arrival) |
| **Departed from South America:**  ***Day of Year*** | DEPART_SA_DoY <-lm(DoY ~ Gen1 + Gen2 + Gen3 + Sex +Latitude + DoY_Spring_Stage_Departure + Lat_Spring_Stage_Departure + DoY_Depart_Winter + Total_Stopover_SA_spring + Avg_Temp_Stage_spring + Avg_MSL_Stage_spring + Avg_TP_Stage_spring +Sex x Latitude + Sex x DoY_Spring_Stage_Departure + Sex x Lat_Spring_Stage_Departure + Sex x DoY_Depart_Winter + Sex x Total_Stopover_SA_spring + Sex x Avg_Temp_Stage_spring + Sex x Avg_MSL_Stage_spring + Sex x Avg_TP_Stage_spring) |
| **Departed from South America:**  ***Latitude*** | DEPART_SA_LAT <-lm(Latitude ~ Gen1 + Gen2 + Gen3 + Sex +DoY + Total_Stopover_SA_spring + DoY_Spring_Stage_Departure + DoY_Depart_Winter + Lat_Spring_Stage_Departure + Avg_Temp_Stage_spring + Avg_MSL_Stage_spring + Avg_TP_Stage_spring +Sex x DoY + Sex x Total_Stopover_SA_spring + Sex x DoY_Spring_Stage_Departure + Sex x DoY_Depart_Winter + Sex x Lat_Spring_Stage_Departure + Sex x Avg_Temp_Stage_spring + Sex x Avg_MSL_Stage_spring + Sex x Avg_TP_Stage_spring) |
| **Arrived at Breeding Grounds:**  ***Day of Year*** | BREEDING_ARRIVAL_DoY <-lm(DoY ~ Gen1 + Gen2 + Gen3 + Sex + Total_Stopover_NA_Spring+ DoY_SA_Departure + DoY_Winter_Departure + Latitude + MSL_SA_Departure + Temp_SA_Departure + TP_SA_Departure) + Sex x Total_Stopover_NA_Spring+ Sex x DoY_SA_Departure + Sex x DoY_Winter_Departure + Sex x Latitude + Sex x MSL_SA_Departure + Sex x Temp_SA_Departure + Sex x TP_SA_Departure) |
| **Arrived at Breeding Grounds:**  ***Latitude*** | BREEDING_ARRIVAL_LAT <-lm(Latitude ~ Gen1 + Gen2 + Gen3 + Sex + Total_Stopover_NA_Spring+ DoY + DoY_SA_Departure + DoY_Winter_Departure + MSL_SA_Departure + Temp_SA_Departure + TP_SA_Departure+ Sex x Total_Stopover_NA_Spring+ Sex x DoY + Sex x DoY_SA_Departure + Sex x DoY_Winter_Departure + Sex x MSL_SA_Departure + Sex x Temp_SA_Departure + Sex x TP_SA_Departure) |
